# Phytochemical analysis, radical scavenging and glioblastoma U87 cells toxicity studies of stem bark of buckthorn (*Rhamnus pentapomica* R. Parker)

**DOI:** 10.1186/s12906-023-04309-w

**Published:** 2024-01-02

**Authors:** Yaseen Ur Rehman, Arshad Iqbal, Gowhar Ali, Ghallab Alotaibi, Alshebli Ahmed, Muhammad Ayaz

**Affiliations:** 1 Department of Botany, Islamia College Peshawar, Peshawar, Khyber Pakhtunkhwa Pakistan; 2 Department of Pharmacy, University of Peshawar, Peshawar, Khyber Pakhtunkhwa 25120 Pakistan; 3 Department of Pharmaceutical Sciences, College of Pharmacy, Al-Dawadmi Campus, Shaqra University, Shaqra, Kingdom of Saudi Arabia; 4 Public Health Department Health Sciences College at Lieth, Umm Al Qura University, Makkah, Kingdom of Saudi Arabia; 5 Faculty of Public and Environmental Health, UofK, Khartoum, Sudan; 6 Department of Pharmacy, Faculty of Biological Sciences, University of Malakand, Chakdara, Dir (L), KP 18000 Pakistan; 7 Department of Pharmacy, University of Malakand, Dir (L), Khyber Pakhtunkhwa 18800 Pakistan

**Keywords:** *Rhamnus pentapomica*, GC-MS, MTT, Free radicals, Glioblastoma

## Abstract

**Background:**

During the past two decades, the correlation between oxidative stress and a variety of serious illnesses such as atherosclerosis, chronic obstructive pulmonary disease (COPD), Alzheimer disease (AD) and cancer has been established. Medicinal plants and their derived phytochemicals have proven efficacy against free radicals and their associated diseases. The current work was aimed to evaluate the phytochemical constituents of *Rhamnus pentapomica* R. Parker via Gas Chromatography-Mass Spectrometry (GC–MS) and its antioxidant and anti-glioblastoma potentials.

**Methods:**

The bioactive compounds were analysed in *Rhamnus pentapomica* R. Parker stem bark extracts by GC–MS analysis, and to evaluate their antioxidant and anti-glioblastoma effects following standard procedures. The stem bark was extracted with 80% methanol for 14 days to get crude methanolic extract (Rp.Cme) followed by polarity directed fractionation using solvents including ethyl acetate, chloroform, butanol to get ethyl acetate fraction (Rp.EtAc), chloroform fraction (Rp.Chf) and butanol fraction (Rp.Bt) respectively. Antioxidant assay was performed using DPPH free radicals and cell viability assay against U87 glioblastoma cancer cell lines was performed via MTT assay.

**Results:**

In GC-MS analysis, thirty-one compounds were detected in Rp.Cme, 22 in Rp.Chf, 24 in Rp.EtAc and 18 compounds were detected in Rp.Bt. Among the identified compounds in Rp.Cme, 9-Octadecenoic acid (Z)-methyl ester (7.73%), Octasiloxane (5.13%) and Heptasiloxane (5.13%), Hexadecanoic acid, methyl ester (3.76%) and Pentadecanoic acid, 14-methyl-, methyl Ester (3.76%) were highly abundant.. In Rp.Chf, Benzene, 1,3-dimethyl- (3.24%) and in Rp.EtAc Benzene, 1,3-dimethyl-(11.29%) were highly abundant compounds. Antioxidant studies revealed that Rp.Cme and Rp.EtAc exhibit considerable antioxidant potentials with IC_50_ values of 153.53 μg/ml and 169.62 μg/ml respectively. Both fractions were also highly effective against glioblastoma cells with IC_50_ of 147.64 μg/ml and 76.41ug/ml respectively.

**Conclusion:**

Phytochemical analysis revealed the presence of important metabolites which might be active against free radicals and glioblastoma cells. Various samples of the plant exhibited considerable antioxidant and anti-glioblastoma potentials warranting further detailed studies.

**Supplementary Information:**

The online version contains supplementary material available at 10.1186/s12906-023-04309-w.

## Introduction

*Rhamnus pentapomica* R. Parker a medicinal plant of the family Rhamnaceae, is a sizable genus with 160 species found all over the world [1]. It is widely distributed in Afghanistan, India and various regions of Pakistan including Orakzai Khyber Pakhtunkhwa, Rakhni, Barkhan and Baluchistan [2]. In Pakistan, its distribution is mostly observed in Khyber Pakhtunkhwa and Baluchistan’s geographic regions [2, 3]. The word “rhamnus” comes from the Ancient Greek word “rhámnos” which is used to designate a number of prickly plants, a thorny bush, or small trees, also known as buckthorn [4]. It was found that *Rhamnus* exhibited antioxidant and anti-proliferative potentials [5]. *Rhamnus kurdica* and *Rhamnus alaternus* have been reported to possess anti-oxidant potentials [6] while *Rhamnus frangula* extract exhibit tumour-inhibitory properties [7].

Reactive oxygen species (ROS) if not properly scavenged by immune system antioxidants, can readily attack various biomolecules like proteins, lipids, lipoproteins, DNA and cause oxidative harm to neuronal cell nuclei and mitochondrial DNA [8, 9]. Oxidative stress is a warning that a cell’s capacity for antioxidant defence is being overwhelmed by the destructive generation of reactive oxygen and nitrogen species (RONS) [10]. These RONS species have a significant role in neurodegenerative disease pathology, deterioration of food, ageing and an increase in malignant cells, autoimmune and inflammatory diseases, can disrupt cellular building blocks and cellular signalling [11, 12]. Many serious health problems like cardiovascular diseases, rheumatism, diabetes mellitus, arteriosclerosis and cancer have been recognised as due to the ROS factors [13–15]. Cancer is a significant global issue because it is the second largest cause of death in underdeveloped countries and the first cause in prosperous nations [16, 17]. Even though there are effective treatment options for the early stages of cancer, such as chemotherapy, surgery, radiotherapy, and hormone therapy. Their use is expensive and is seldom used in the latter stages of the disease [18]. Glioblastoma, which accounts for 80% of all malignant brain tumours, is the most prevalent type of adult brain tumour and has the most aggressive and treatment-resistant forms [19, 20]. The most common and dangerous subtype is glioblastoma, a grade 4 astrocytic tumour specified by the World Health Organization (WHO). It has a high rate of growth and distant metastasis, making it resistant to conventional temozolomide chemotherapy, surgical excision, and local radiation therapy. Despite cutting-edge treatments like radiation therapy, glioma has a relatively low healing rate and a disappointing 5-year survival rate [21]. To improve patients’ overall outlooks, it is necessary to find innovative drugs that can alter the blood-brain barrier (BBB), decrease tumour growth, and stop the development of recurring tumours.

Natural products are being investigated by researchers as potential second-line therapies alternative to chemotherapy or even as chemo preventative medications [22]. Many studies have shown the critical role of the plant extracts in development of new anticancer drugs [23, 24]. The ability of phenolic compounds to scavenge free radicals has been established [25]. These compounds work by chelating metal ions, reducing the generation of free radicals, and boosting the body’s natural antioxidant system [26, 27]. They can donate with hydrogen or electrons and still remain stable radical intermediates. Polyphenols, vinca alkaloids, taxanes, epipodophyllotoxins, and camptothecin are just few examples of the plant-derived chemicals that are at the core of the therapeutic formulations used to target particular types of cancer. Natural plant compounds’ multi-factorial qualities can help target particular chemotherapeutic medications, which frequently increase cancer cells’ resistance [28]. Recent research has revealed that the genus *Rhamnus* contains a variety of bioactive substances, including phenolic compounds, alkaloids, steroids, terpenoids, saponins, and glycosides, which have a variety of biological effects [29]. *Rhamnus alaternus* leaves contain triglycoside flavonoids, which have antigenotoxic properties and fight free radicals to keep them from damaging cells [30]. Phenols and flavonoids detected in *Rhamnus cathartica* have strong antioxidant potentials [31]. The objective of this study was to examine the bioactive substances by GC-MS, as well as the antioxidant and anti-glioblastoma properties of *R. pentapomica* R. Parker.

## Materials and methods

### Chemicals, reagent and cell lines

Methanol, *n*-Hexane, Chloroform, Ethyl acetate, n- Butanol were purchased from Sigma-Aldrich Chemical (*S.Q.F*, *France*), purified water, DPPH, vitamin C and DMSO were taken from Department of Pharmacy University of Peshawar, Pakistan. The U87 glioblastoma cells line, Dulbecco’s modified Eagle’s medium (DMEM), fatal bovine serum (FBS), penicillin–streptomycin, MTT reagent and Doxorubicin (Sigma-Aldrich) were acquired from Khyber Medical University, Hayat Abad Peshawar, Pakistan.

### Plant collection and authentication

The stem bark of *Rhamnus pentapomica* R. Parker was collected from the hills of Khee Kada, District Orakzai, Khyber Pakhtunkhwa, Pakistan. The plant specimen was taken to the Herbarium of the Department of Botany, Islamia College Peshawar and was authenticated by Dr Gulam Jilane, Lecturer Department of Botany, University of Peshawar, and Dr Naveed Akhtar, Associate Professor Department of Botany, Islamia College Peshawar, Pakistan. After identification, the specimen was given voucher number (Yas-ICP-322) and was placed in Herbarium Department of Botany, Islamia College Peshawar for future reference.

### Crude extract preparation and fractionation

The collected plant was cleaned by washing and rinsing with distilled water to remove any adulterants. Subsequently, it was subjected to shade drying for 22–25 days with random checking. The dried plant material was converted to powder form using an electric grinder and passed through sieve number 30. 1 kg of powdered material was macerated in 80% methanol (05 L) for 14 days at room temperature with occasional shaking. The solvent was removed and again same amount of solvent was added (05-L × 3) to dissolve the phytochemicals completely. All solvents obtained were combined, filtered through muslin cloth and then through filter paper. The extract was concentrated using rotary evaporator under reduce pressure to get 150 g (15%) of crude extract (Rp.Cme). From the extracted Rp.Cme, 120 g was suspended in about 500 ml of distilled water and partitioned in separating funnel with equal volume of ethyl acetate (500 ml × 3), chloroform (500 ml × 3) and butanol (500 ml × 3). The percent yields for various fractions were Rp.EtAc 22 g (18.33%), Rp.Chf 17 g (14.16%) and Rp.Bt 9.5 g (3.3%). All samples were kept in air tight glass bottles and were refrigerated till further use [32].

### Gas Chromatography Mass Spectrometry Analysis (GC–MS)

Gas Chromatography Mass Spectrometry (GC-MS) was used to assess the phytochemical composition of the samples. It involves loading of the sample onto the GC column, followed by separation of its ingredients onto the analytical column. Following previously reported procedure; 10 mg of the desired samples were dissolved in 5 ml of the respective solvent and was diluted by adding 9 ml of solvent to 1 ml sample. Samples analysis was performed using the GC-MS-QP2010 instrument’s Db. 30.0 column, which has a 0.25 mm diameter and 0.25 mm thickness (SHIMADZU instrument). A carrier gas of helium was used with a flow rate of 1 ml/min, a pressure of 99.99 percent, an injector volume of 2 ul, and a temperature of 280 °C. The oven’s temperature was gradually increased from 40 to 280 °C for 5 min. The oven was set to heat up from 70 degrees (isothermal for 5 min) to 200 degrees (10 degrees per minute), then 5 degrees per minute, up to 280 degrees, then isothermal at 280 degrees for 35 min. At 70 eV, 0.5 s scans intervals, and a scan range of 40–1000 m/z, mass spectra were acquired. Samples were automatically injected with electronic pressure control after being fully dissolved in the relevant solvent. Based on retention time and MS fragment ions, the bioactive chemicals were identified, and their ratio to the total peak area was calculated. By correlating the MS spectrum patterns of the phytochemicals with the standard mass spectra found in the National Institute of Standards and Technology (NIST) Mass Spectra Database, the phytochemicals were identified [33].

### DPPH anti-radicals assay

For measuring the antioxidant potentials of the plant samples, DPPH (1, 1-diphenyl-2-picrylhydrazyl) free radicals scavenging assay was used as we reported previously [34, 35]. Briefly, fresh working solution of DPPH was prepared by dissolving 4 ml (0.004 g) of DPPH reagent powder into 100 ml of methanol (0.004 percent). Before use, the working solution was stored in a brown bottle in dark place to produce DPPH radicals. Samples were dissolved in respective solvents at 1 mg/ml concentration and serial dilutions were prepared including 100 µg/ml, 200 µg/ml, 400 µg/ml, 800 µg/ml and 1000 µg/ml of each of these 4 stock solutions of each fraction were prepared. Thereafter, 50 µl of 0004% (4 mg/ml) of the DPPH solution was added to 50 µl of each sample solutions in the test tubes. Test tubes were maintained in dark for 30 min. For the preparation of the negative control, 50 µl of methanol and 2 ml of DPPH solution were used, while the preparation of the positive control, 50 µl of ascorbic acid and 2 ml of DPPH solution were used. The radical scavenging activity was determined by a drop in DPPH absorbance. The mixtures were tested for antioxidant activity at the conclusion of the incubation time using an Optima UV-Visible spectrophotometer at a wavelength of 517 nm. The following formula was used to calculate the anti-radical potentials of each sample.

$$\mathrm{DPPH\, scavenging\, }\left(\mathrm{\%}\right)=\frac{\mathrm{Control\, }\left({\text{Mabs}}\right) -\mathrm{Extract\,}({\text{Rabs}})}{\mathrm{Control\, }({\text{Mabs}})}\times 100$$

Where M_abs_ represents negative control (Methanol) absorbance and Rabs indicates samples absorbance. The percent inhibition values were used to calculate IC_50_ values of the samples.

### Antitumor study using glioblastoma U87 cells

#### Sample preparation

From each sample including Rp.Cme, Rp.Chf, Rp.EtAc, and Rp.Bt 1 mg was separately dissolved in 1 ml of 0.1% DMSO to make the stock solution. It was then added to the culture media in accordance with the Dulbecco’s modified Eagle’s medium (DMEM) procedure [36].

#### Cell culture

Incubated at 37 °C in a humidified incubator with 5% Carbon dioxide and 95% air, glioma U87 cells were grown in Dulbecco’s modified Eagle’s medium (DMEM), which contains 10% foetal bovine serum (FBS) and 1% penicillin-streptomycin.

#### Cell viability assay

Maximum 7 × 103 cells were cultivated in 100 L of Dulbecco’s modified Eagle’s medium (DMEM), 10% FBS, streptomycin, and penicillin in 96-well microplates after becoming trypsinized and counted by Neubauer chamber. Cells were the incubated at 37 °C, 5% CO_2_ for 24 h. The cells were permitted to adhere to the plate’s bottom in order to obtain an acceptable number of cells per plate. Inverted microscope was used to continuously assess the cell count and growth quality. The extra fluid was then disposed. U87 glioma cells were exposed to samples including Rp.Cme, Rp.Chf, Rp.EtAc, and Rp.Bt at concentrations ranging from 7.81–500 µg/ml for 24 h. The U87 cells were first planted in 96-well plates and subsequently given the aforementioned treatments. After 24 h, the culture medium was removed, and each well received 25 L of FBS containing 0.25 mg/ml of MTT reagent (5 mg/ml). The MTT reagent-containing culture medium was cultured for 24 h at 37 °C with 5% CO_2_. Insoluble formazan crystals were dissolved in DMSO after the medium containing the MTT reagent was removed, and then the absorbance of each well was measured at 630 nm using a spectrophotometer. Experiments were performed in triplicate. The following formulae were used to determine the percentages of cell survival and inhibition;

$$\begin{array}{l}\mathrm{Cell\, survival\, }(\mathrm{\%}) =\frac{\mathrm{Absorbance\, of\, test\, sample}}{\mathrm{absorbance\, of\, the\, negative\, control}}\times 100\\ \mathrm{\%\, inhibition\, of\, cell\, viability} =\frac{\mathrm{Control }-\mathrm{ treatment}}{{\text{Control}}}\times 100\end{array}$$

### Statistical analysis

All results were presented as Mean ± SEM of three independent experimental observations. For the results analysis of GC-MS, GC Shimadzu software was applied. Data was analysed using SPSS software for statistical analysis. One-way ANNOVA followed by multiple comparison Dunnett’s test were applied for the comparison between test group and control. *P* values < 0.05 were considered as statistically significant. The IC_50_ numbers were set up as treatment concentrations bring about a 50% reduction in the viability or reducing the bioactivity.

## Results

### GC-MS analysis of the samples

**Fig. 1:**
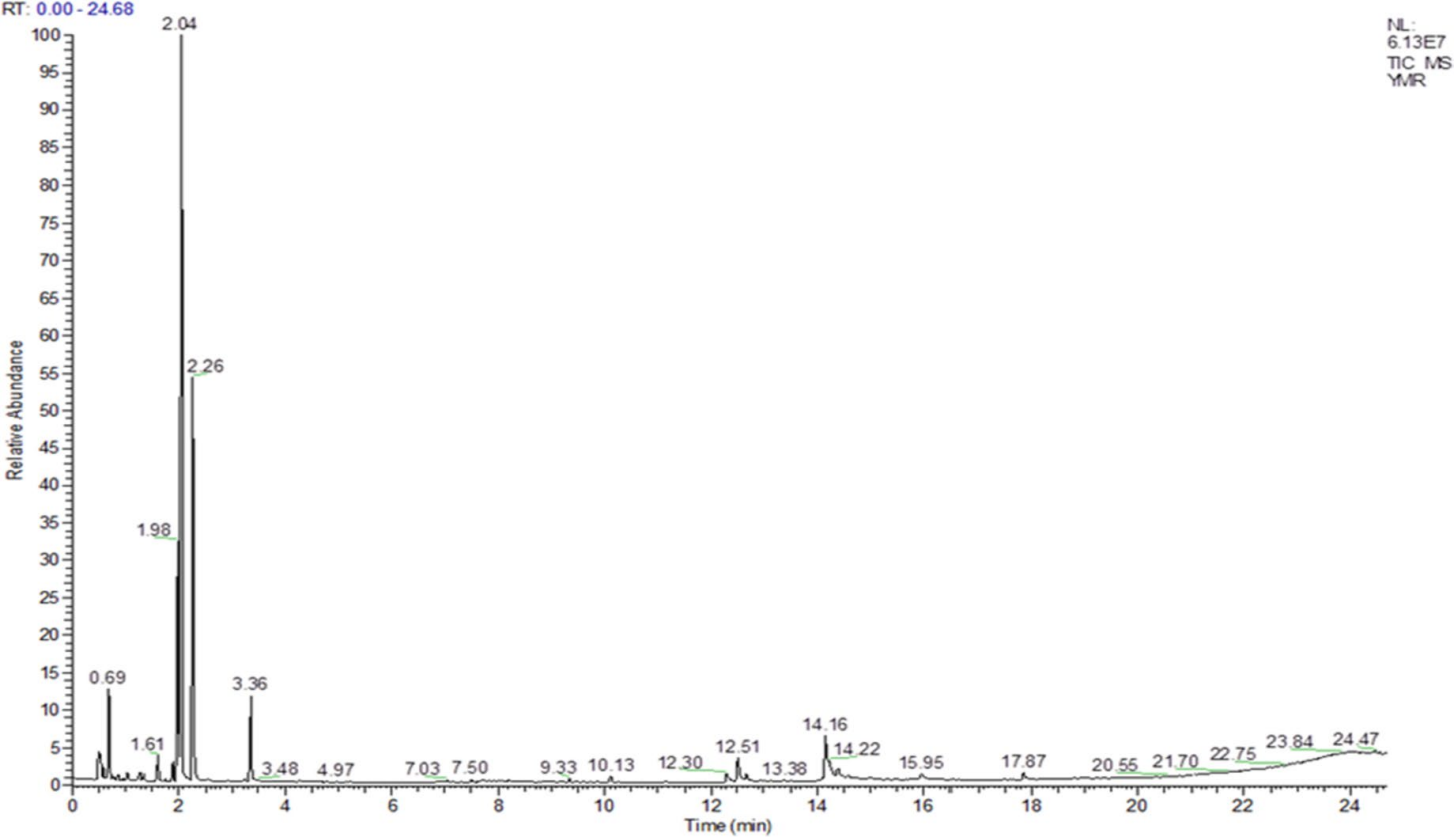
GC-MS chromatogram of Rp.Cme

**Fig. 2:**
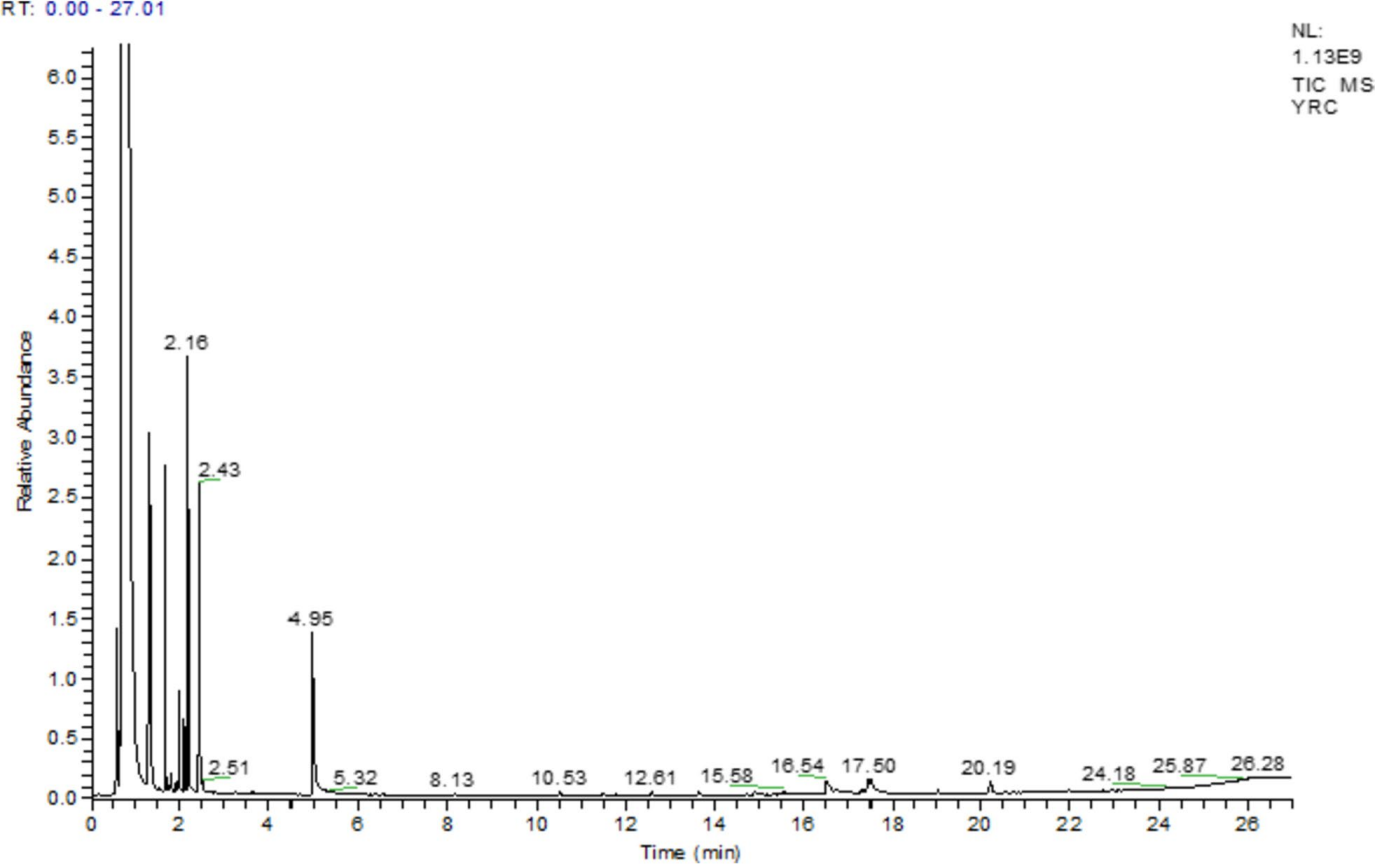
GC-MS chromatogram of Rp.Chf

**Fig. 3:**
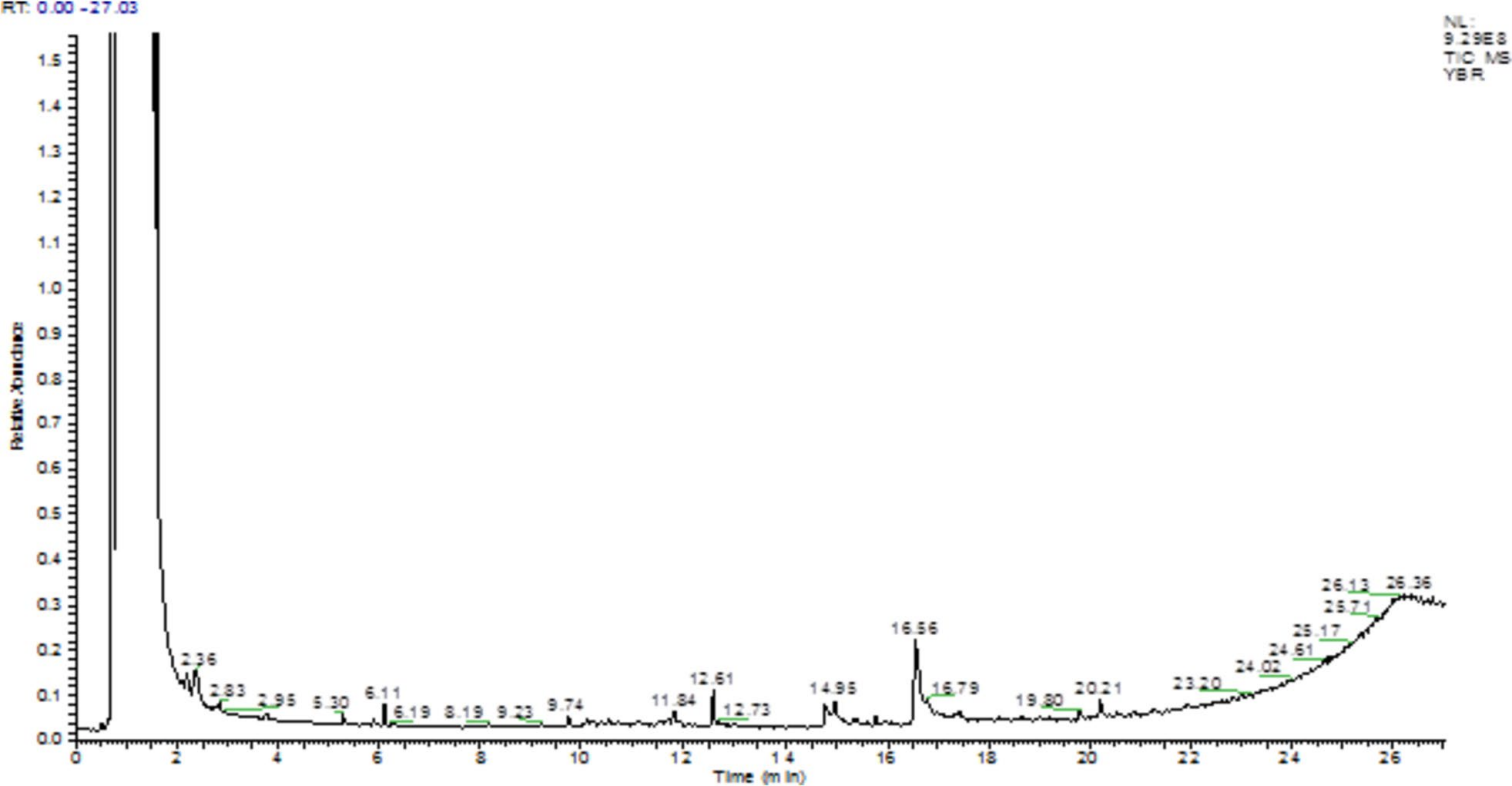
GC-MS chromatogram of Rp.EtAc

**Fig. 4:**
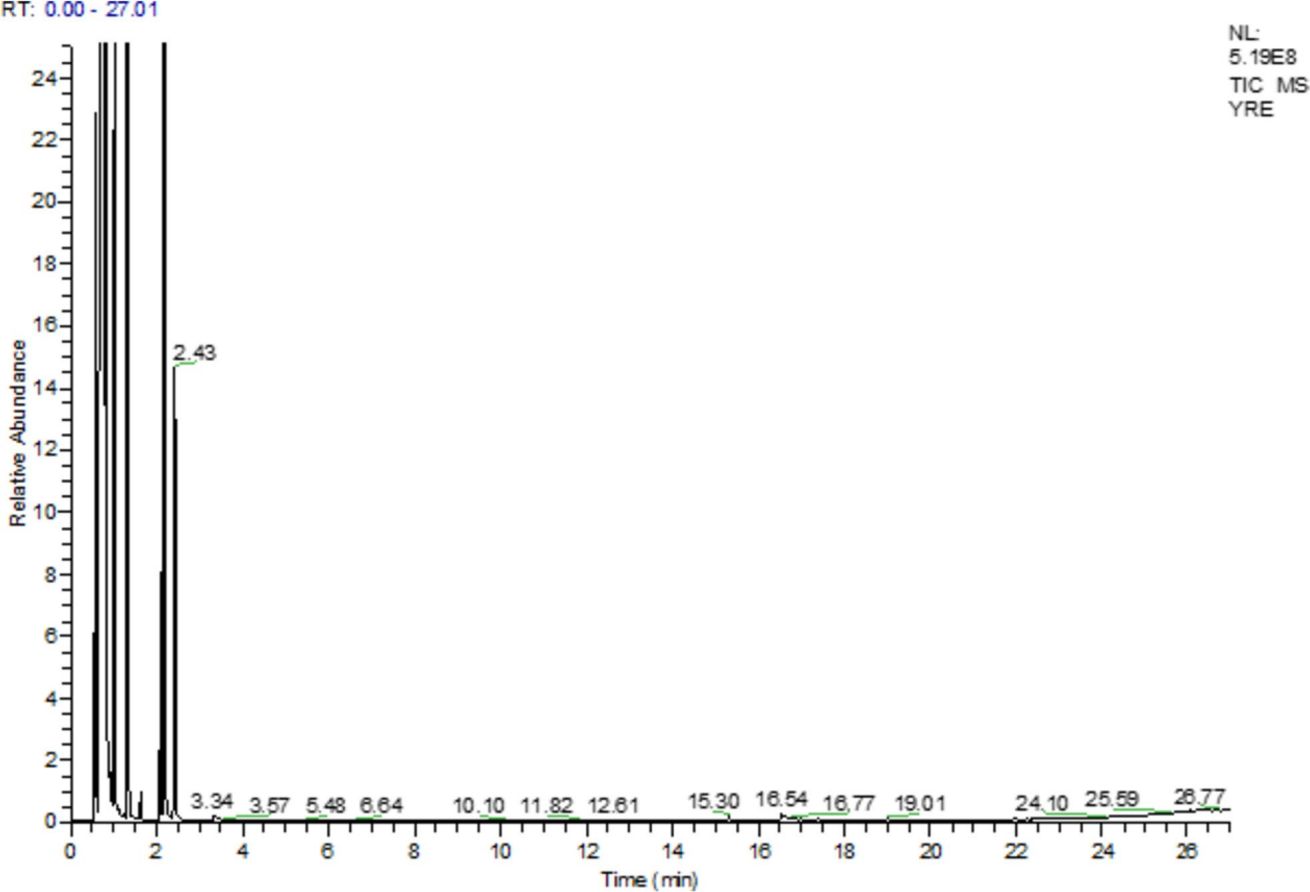
GC-MS chromatogram of Rp.Bt

GC–MS profiling resulted the identification of several phytochemicals in *Rhamnus Pentapomica* R. Parker extracts as indicated by their molecular formula and retention time. The Rp.Cme revealed the presence of 31 compounds. Likewise, Rp.Chf, Rp.EtAct and Rp.Bt revealed the presence of 22, 24 and 18 compounds respectively (Tables S1 to S4). The mass spectrum of the reported compounds is given as Figs. 1, 2, 3 and 4.

Among the identified compounds in the GC-MS analysis of Rp.Cme, 9-Octadecenoic acid, methyl ester, (E) (7.73%), 9-Octadecenoic acid (Z)-methyl ester (7.73%), 12-Octadecenoic acid, methyl ester (7.73%), octasiloxane, 1,1,3,3,5,5,7,7,9,9,11,11,13,13,15,15-hexadecamethyl (5.13%), hexasiloxane, 1,3,3,5,5,7,7,9,9,11,11 dodecamethyl (5.13%), Heptasiloxane,1,1,3, 3,5,5,7,7,9,9,11,11,13,13-tetradeca methyl (5.13%), cyclohexane, 1,1-dimethoxy (4.13%), 3-Oxo-4-methylpentanoic acid, methyl ester, enol form (4.13%), trans 3-methyl-2-n-propylthiophane (4.13%) were highly abundant compounds. Among the other identified compounds, hexadecanoic acid, methyl ester (1.16%), pentadecanoic acid, 14-methyl-methyl ester (1.16%), hexadecanoic acid, methyl ester (3.76%), acetic acid-butyl ester (1.16%), octadecanoic acid, 9-oxo-, methyl ester (1.05%), octadecanoic acid, 10-oxo-methyl ester1 (1.05%), and oleic acid, eicosyl ester (0.03%) were present in comparatively low concentrations.

Likewise, in GC-MS analysis of Rp.Chf, 22 compounds were identified as shown in Table S2 and Fig. 2. Among these, benzene, 1,3-dimethyl-(3.24%), benzene, nitro-(1.26%), 8-octadecenoic acid, methyl ester (0.32%), 9-octadecenoic acid, methyl ester (0.32%), 9-octadecenoic acid (Z)-methyl ester (0.32%) were present in comparatively high concentrations. Further, 24 compounds were identified in Rp.EtAc as shown in Table S3 and Fig. 3. Among these, benzene, 1, 3-dimethyl (11.29%), 9-octadecenoic acid (Z)-methyl ester (0.34%), 9-octadecenoic acid, methyl ester (0.34%), 8-octadecenoic acid, methyl ester (0.34%), octasiloxane, 1,1,3,3,5,5,7,7,9,9,11,11,13,13,15,15-hexadecamethyl (0.33%), hexasiloxane, 1,1,3,3,5,5,7,7,9,9,11,11-dodecamethyl (0.33%), heptasiloxane, 1,1,3,3,5,5,7,7,9,9,11,11,13,13-tetradecamethyl (0.33%) were highly abundant compounds.

A total of 18 compounds were found in Rp.Bt as shown in Table S4 and Fig. 4. Among these, hexasiloxane, 1,1,3,3,5,5,7,7,9,9,11,11-dodecamethyl (0.09%), octasiloxane, 1,1,3,3,5,5,7,7,9,9,11,11,13,13,15,15-hexad-ecamethyl (0.09%), heptasiloxane, 1,1,3,3,5,5,7,7,9,9,11,11,13,13-tetradeca methyl (0.09%), 8-octadecenoic acid, methyl ester (0.06%), 9-octadecenoic acid, methyl ester (0.06%), 11-octadecenoic acid, methyl ester, (Z) (0.06%), pentadecanoic acid, 14-methyl-, methyl ester (0.02%), hexadecanoic acid, methyl ester (0.02%), hexadecanoic acid, methyl ester (0.02%), 3-hexanone, 2,5-dimethyl-4-nitro (0.01%), pentanoic acid, and 2, 2, 4-trimethyl-3-hydroxy-isobutyl ester (0.01%) were the identified compounds.

### Anti-oxidant study

**Fig. 5:**
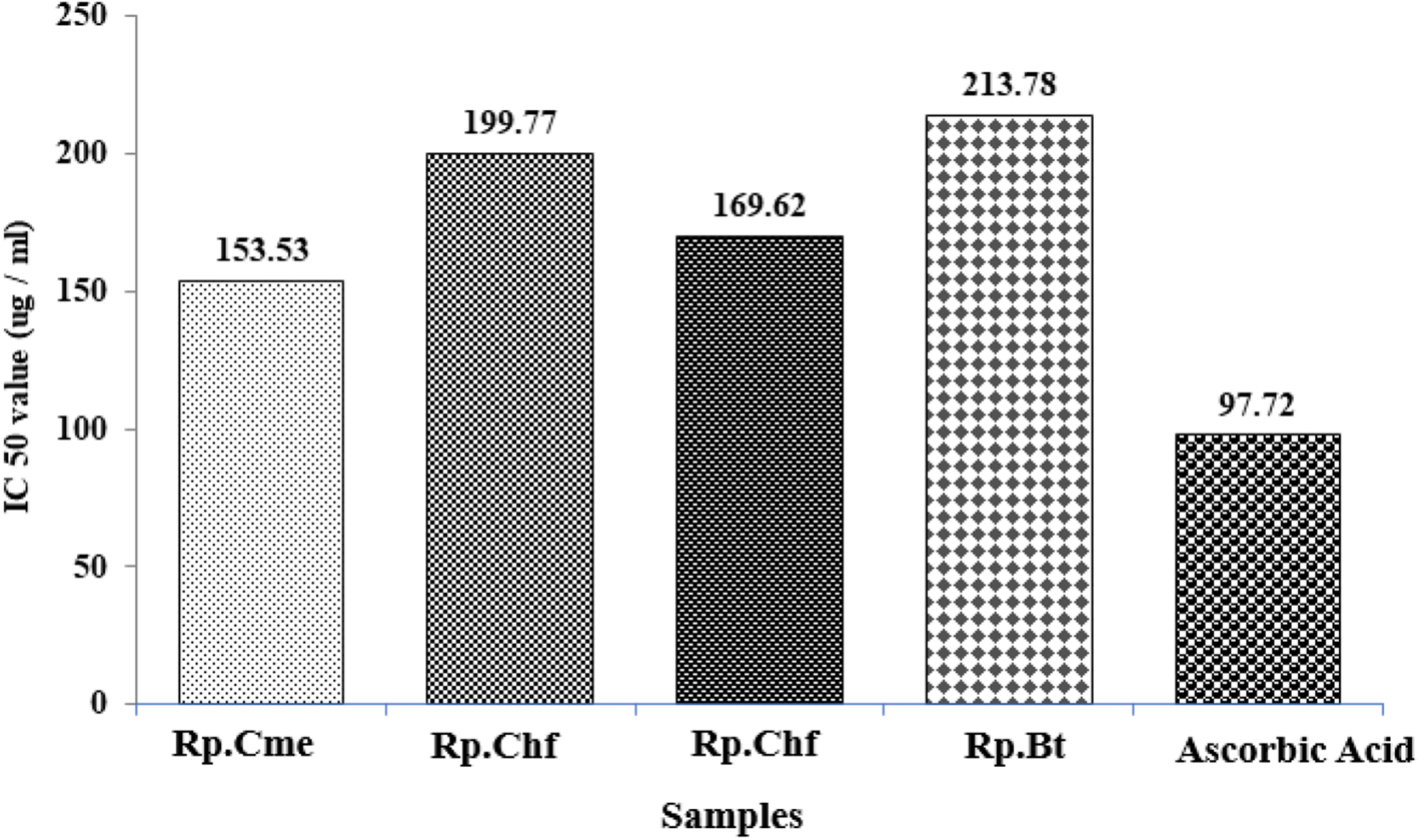
The IC_50_ values of DPPH free radical scavenging assay for various samples

Samples extracted from *Rhamnus pentapomica* R. Parker exhibited considerable antioxidant potential as shown in Table 1 and Fig. 5. Rp.Cme revealed considerable concentration dependent scavenging effect against free radicals i.e. 40.42% percent at 100 µg/ml, 54.38% at 200 µg/ml, 77.25% at 400 µg/ml, 84.2% at 800 µg/ml and 89.36% at 1000 µg/ml. Furthermore, Rp.Chf demonstrated considerable anti-radicals activity (33.33%, 49.42%, 67.81%, 80.45%, and 83.90%) at 100, 200, 400, 800, and 1000 µg/ml respectively. The Rp.EtAc also exhibited dose-dependent scavenging effect (38.70%) at 100 µg/ml, (54.83%) at 200 µg/ml, (66.66%) at 400 µg/ml, (76.34%) at 800 µg/ml, and (84.5%) at 1000 µg/ml at 100, 200, 400, 800, and 1000 µg/ml concentrations respectively. The Rp.Bt also showed (33.70%), (48.31%), (64.83%), (73.03%) and (83.14%) scavenging effect at the same tested concentrations. Ascorbic acid caused 55.31%, 60.63%, 75.53%, 87.23% and 93.61% inhibition of the free radicals at the same concentrations respectively. The Rp.Cme was highly potent among the sample with IC_50_ value of 153.53 µg/ml, whereas other samples Rp.EtAc (IC_50_ value 169.62 µg/ml), Rp.Chf (IC_50_ 199.77 µg/ml) and Rp.Bt (IC_50_ values of 213.78 µg/ml) were also highly active. Ascorbic acids revealed IC_50_ value of 97.72 µg/ml.

**Table 1:** DPPH free radical scavenging effect of *Rhamnus Pentapomica* R. Parker

**S. No**	**Conc. µg/ml**	**Scavenging % ± SEM (*n* = 3)**				
		**Rp.Cme**	**Rp.Chf**	**Rp.EtAc**	**Rp.Bt**	**Ascorbic acid**
**1**	100	40.42 ± 1. 20^ ns^	33.33 ± 0. 59**	38.70 ± 1.60*	33.70 ± 0.99**	55.31 ± 0.05
**2**	200	54.38 ± 1. 36^ ns^	49.42 ± 0.89^ ns^	54.83 ± 0.28^ ns^	48.31 ± 0.81^ ns^	60.6 3 ± 0.01
**3**	400	74.25 ± .1.16^ ns^	67.81 ± 0. 95^ ns^	66.66 ± 0.12^ ns^	64.83 ± 1.34^ ns^	75.53 ± 0.02
**4**	800	84.25 ± 1.97^ ns^	80.45 ± 0.82^ ns^	76.34 ± 1.87^ ns^	73.03 ± 1.43^ ns^	87.23 ± 0.001
**5**	1000	89.36 ± 1.20^ ns^	83.90 ± 0.18^ ns^	84.51 ± 1.12^ ns^	83.14 ± 2.14^ ns^	93.61 ± 0.05

*Rp.Cme* Methanol extract, *Rp.Chf* Chloroform, *Rp.EtAc* Ethyl acetate, *Rp.Bt* Butanol fractions, *ns* Values not significantly different when compared with standard/control group

^*^p < 0.05 and **p < 0.01 when compared with standard group

### Anti-glioblastoma study

**Fig. 6:**
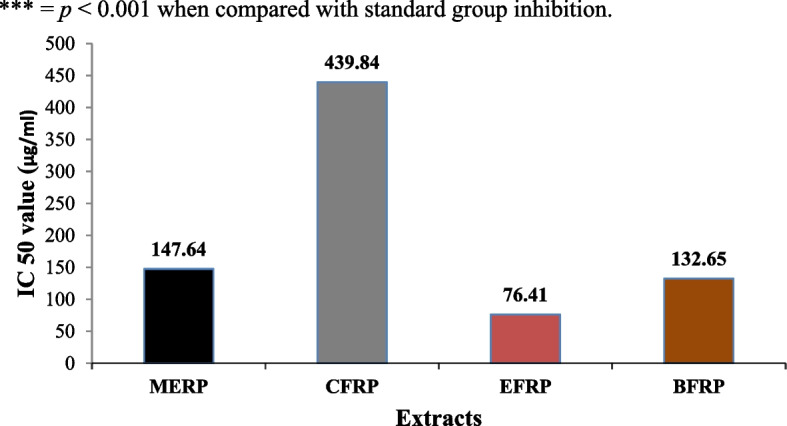
The IC_50_ values for Rp.Cme, Rp.Chf, Rp.EtAc and Rp.Bt in the antitumor assays

**Fig. 7:**
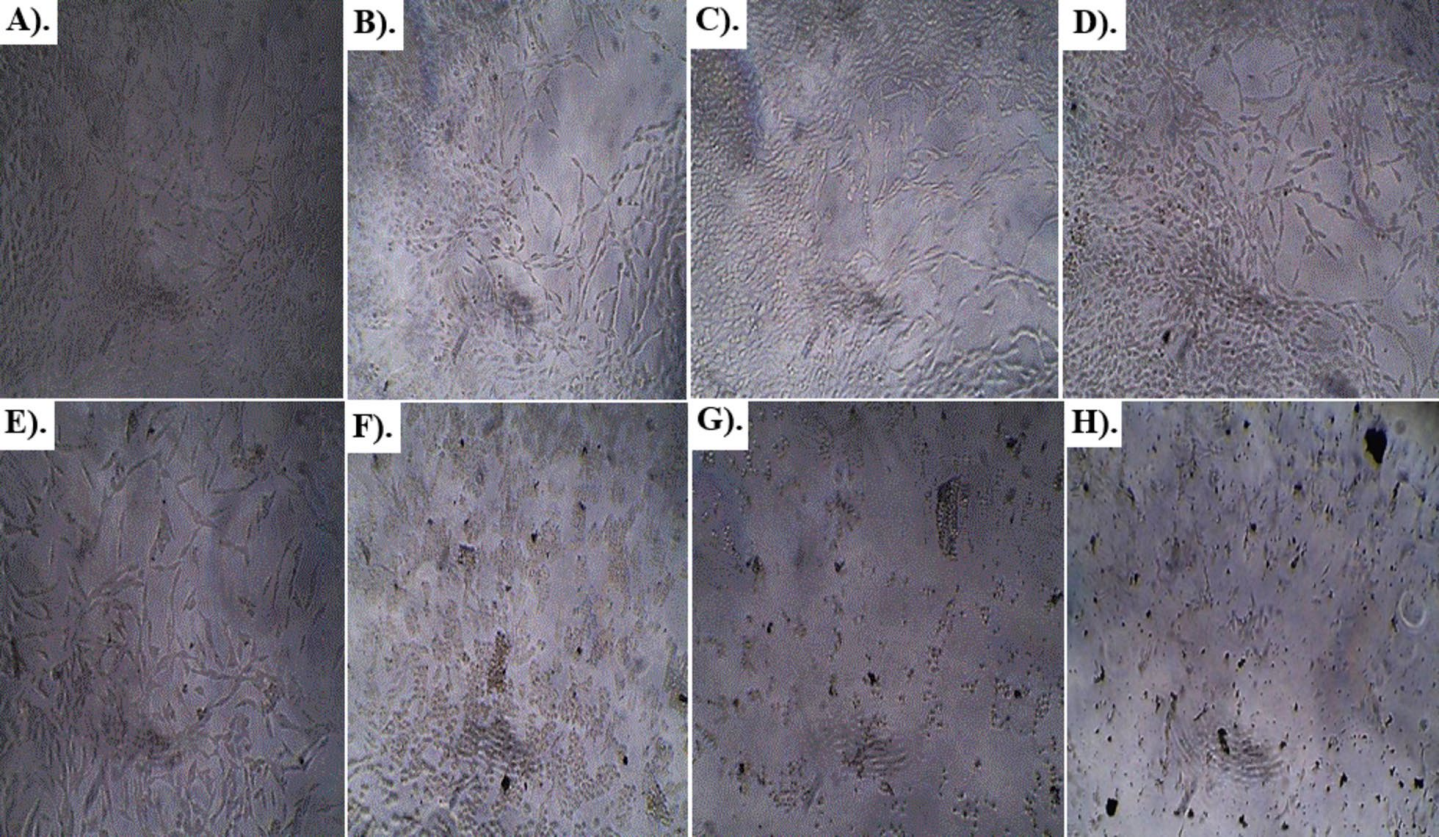
Morphological transforms observed in U87 glioblastoma cancer cells treated with different concentrations (7.81, 15.62, 31.25, 62.5, 125, 250 and 500 µg/ml) of Rp.Cme for 24 h. Cells treated with 125, 250 and 500 µg/ml (**F**, **G**, **H**) of Rp.Cme for 24 h showed the greatest morphological changes compared with the control cells (**A**). Pictures were taken after 24 h by optic microscopy. **A** Untreated cells. **B** Cells treated with 7.81 µg/ml Rp.Cme. **C** Cells treated with 15.62µg/ml Rp.Cme. **D** Cells treated with 31.25µg/ml Rp.Cme. **E** Cells treated with 62.5µg/ml Rp.Cme. **F** Cells treated with 125 µg/ml Rp.Cme. **G** Cells treated with 250 µg/ml Rp.Cme. **H** Cells treated with 500 µg/ml Rp.Cme

**Fig. 8:**
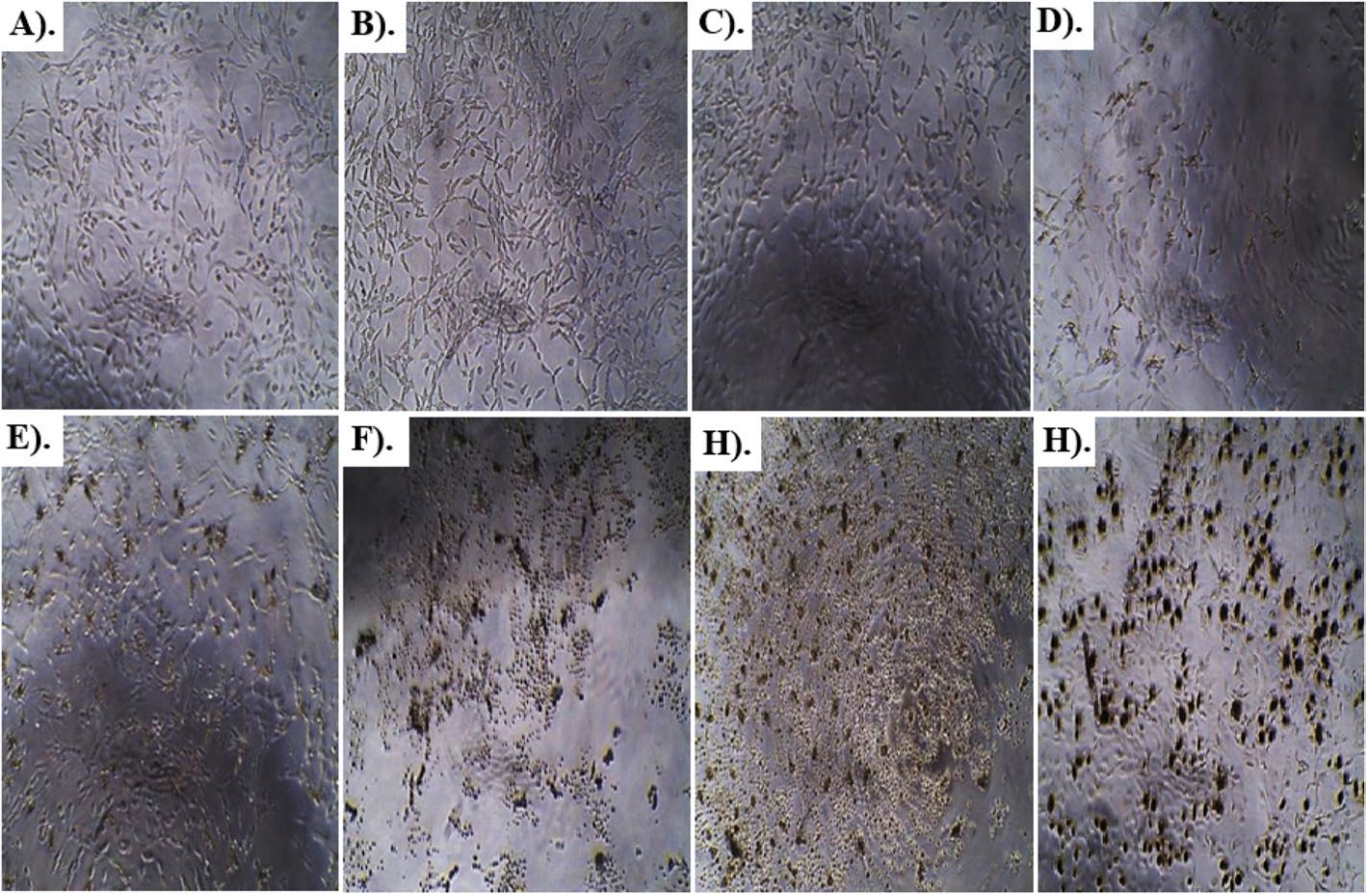
Morphological transforms observed in U87 glioblastoma cancer cells treated with different concentrations of Rp.Chf for 24 h. The cell viability assay was performed to measure glioblastoma U87 cells viability after treatment with increasing concentrations of Rp.Chf. Cells treated with 31.25, 62.5, 125, 250 and 500 µg/ml (**D**, **E**, **F**, **G**, **H**) of Rp.Chf for 24 h showed the greatest morphological changes compared with the control cells (**A**). Pictures were taken after 24 h by optic microscopy. **A** Untreated cells. **B** Cells treated with 7.81 µg/ml Rp.Chf. **C** Cells treated with 15.62µg/ml Rp.Chf. **D** Cells treated with 31.25µg/ml Rp.Chf. **E** Cells treated with 62.5µg/ml Rp.Chf. **F** Cells treated with 125 µg/ml Rp.Chf. **G** Cells treated with 250 µg/ml Rp.Chf. **H** Cells treated with 500 µg/ml Rp.Chf

**Fig. 9:**
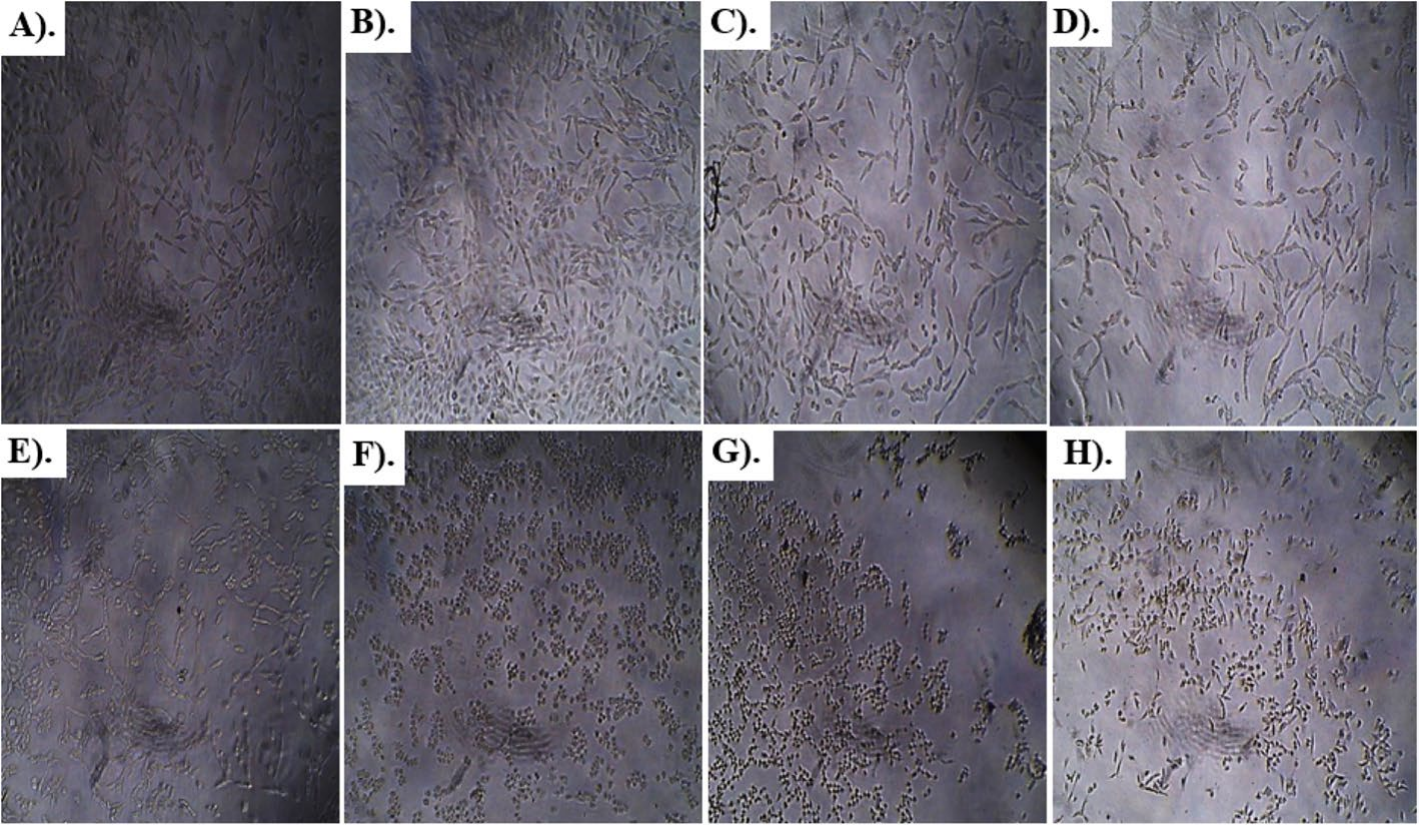
Morphological transforms observed in U87 glioblastoma cancer cells treated with different concentrations of Rp.EtAc for 24 h. Cells viability assay was performed at increasing concentrations of Rp.EtAc (7.81, 15.62, 31.25, 62.5, 125, 250 and 500 µg/ml) for 24h. The cells treated with 62.5, 125, 250 and 500 µg/ml (**E**, **F**, **G**, **H**) of Rp.EtAc for 24 h showed the greatest morphological changes compared with the control cells (**A**). Pictures were taken by optic microscopy. **A** Untreated cells. **B** Cells treated with 7.81 µg/ml Rp.EtAc. **C** Cells treated with 15.62 µg/ml Rp.EtAc. **D** Cells treated with 31.25 µg/ml Rp.EtAc. **E** Cells treated with 62.5 µg/ml Rp.EtAc. **F** Cells treated with 125 µg/ml Rp.EtAc. **G** Cells treated with 250 µg/ml Rp.EtAc. **H** Cells treated with 500 µg/ml Rp.EtAc

**Fig. 10:**
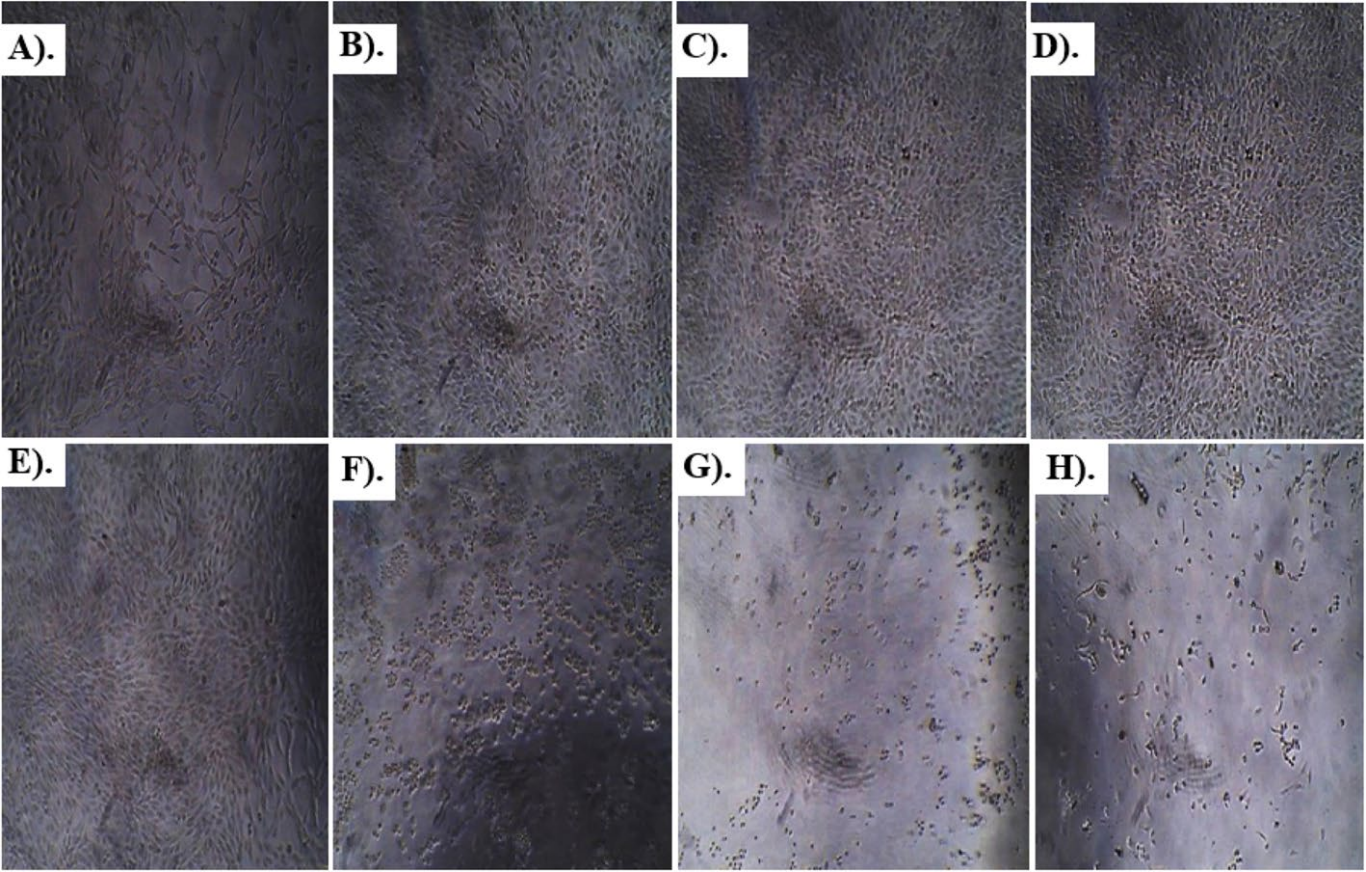
Morphological transforms observed in U87 glioblastoma cancer cells treated with different concentrations of BFRP Rp.Bt for 24 h. Cells viability assay was assessed after treatment with increasing concentrations of Rp.Bt (7.81, 15.62, 31.25, 62.5, 125, 250 and 500 µg/ml) for 24h. **A** Untreated cells. **B** Cells treated with 7.81 µg/ml Rp.Bt. **C** Cells treated with 15.62 µg/ml Rp.Bt. **D** Cells treated with 31.25 µg/ml Rp.Bt. **E** Cells treated with 62.5 µg/ml Rp.Bt. **F** Cells treated with 125 µg/ml Rp.Bt. **G** Cells treated with 250 µg/ml Rp.Bt. **H** Cells treated with 500 µg/ml Rp.EtAc

Results of anti-glioblastoma studies are summarized in Table 2 and Figs. 6, 7, 8, 9 and 10. According to the results, Rp.Cme treatment reduced the viability of cells to 66.13%, 63.19%, 51.4%, 29.61%, 28.28%, 14.14%, and 11.05% at dosage of 500, 250, 125, 62.5, 31.25, 15.62 and 7.81 µg/ml respectively. The Rp.Chf reduces cell viability to 49.53% at 500 µg/ml, 46.65% at 250 µg/ml, 44.41% at 125 µg/ml, 40.1% at 62.5 µg/ml, 38.98% at 31.25 µg/ml, 32.75 at 15.62 µg/ml and 28.28% at 7.81 µg/ml concentrations. The cell viability of the Rp.EtAc was also reduced in a dose-dependent manner with 62.41%, 61.09%, 58.15%, 49.34%, 43.46%, 38.62%, and 24.67% viability at concentrations of 500, 250, 125, 62.5, 31.25, 15.62 and 7 µg/ml. respectively. Cell viability was decreased to 72.38% at 500 µg/ml concentration and further to 60.92, 56.34, 32.35, 23.19, 13.75 and 9.03 percent at 250, 125, 62.5, 31.25, 15.62 and µg/ml correspondingly by treatment with Rp.Bt. Higher anti-glioblastoma activity was seen in the Rp.EtAc fraction (IC_50_ value), which was followed by the Rp.Bt fraction (IC_50_ 132.65 µg/ml) and the Rp.Cme (IC_50_ 147.64 µg/ml (Fig. 6).

**Table 2:** Percent cell survival and inhibition of glioblastoma cell

**S. No.**	**Dose**	**Samples**				
		**Rp.Cme**	**Rp.Chf**	**Rp.EtAc**	**Rp.Bt**	**Standard**
	**Conc. µg/ml**	**Growth Inhibition % ± SEM**	**Growth Inhibition % ± SEM**	**Growth Inhibition % ± SEM**	**Growth Inhibition % ± SEM**	**Growth Inhibition % ± SEM**
**1**	500	66.13 ± 0.01***	49.53 ± 0.004***	62.41 ± 0.0012***	72.38 ± 0.006**	100%
**2**	250	63.19 ± 0.003***	46.65 ± 0.004***	61.09 ± 0.0024***	60.92 ± 0.0097***	100%
**3**	125	51.4 ± 0.003***	44.41 ± 0.005***	58.15 ± 0.0020***	56.34 ± 0.0734***	100%
**4**	62.5	29.61 ± 0.004***	40.1 ± 0.0032***	49.34 ± 0.0061***	32.35 ± 0.0024***	100%
**5**	31.25	28.28 ± 0.003***	38.98 ± 0.004***	43.46 ± 0.0126***	23.19 ± 0.0065***	100%
**6**	15.62	14.14 ± 0. 001***	32.75 ± 0.005***	38.62 ± 0.0057***	13.75 ± 0.0016***	100%
**7**	7.81	11.05 ± 0.01***	28.28 ± 0.012***	24.67 ± 0.0077***	9.03 ± 0.01***	100%

^***^p < 0.001 and ^**^p < 0.01 when compared with standard group inhibition

## Discussion

Medicinal plants and their derived compounds are in use for the management of various diseases since the establishment of human era [37]. With the development of modern analytical techniques, ithas become easy to detect and isolate phytochemical of interest and perform mechanistic studies for potential drug discovery [38, 39]. Since plants are the sources of many effective medications, accurate identification and research of phyto constituents are recurrently evolving. In order to bio prospect for plant bioactive chemicals, it has been established that Gas Chromatography and Mass Spectrometry (GC-MS) is an effective technique. The present research work focused on analysing the major phytochemicals by GC-MS, and to assess the antioxidant and anti-glioblastoma properties of stem bark extracts of *Rhamnus Pentapomica* R. Parker.

Several compounds were identified in Rp.Cme which include 2, 3 bis [(trimethylsilyl) oxy] propyl ester, (Z, Z, Z) which is reported to have antimicrobial and anti-inflammatory potentials [40]. Another important secondary metabolite, oleic acid, eicosyl ester is well known for its anti-inflammatory, cancer preventive, hypocholesterolaemic, antimicrobial and anemiagenic insectifuge [41] potentials. The 2, 3 Dihydroxypropyl elaidate also known as monoelaidin has reported to have antimicrobial, antihypertensive, antioxidant and anticancer properties [42]. Further, 1H-Indene, 2-butyl-5-hexyloctahydro- also has antioxidant, antibacterial, and anti-inflammatory properties and promote wound healing. Yet another bioactive compound 4H-Cyclopropa [5´,6´] benz [1´,2´,7,8] azuleno [5, 6] oxiren-4-one,8,8a- known to possess antibacterial and antioxidant activity [43], whereas another identified compound, 1-hexadecanol is also known for its anti-inflammatory, nematicide, pesticide, lubricant, anti-androgenic, flavour, haemolytic 5-alpha reductase inhibitor, antioxidant, hypocholesterolemic activities [43]. Bioactive compound dasycarpidan-1-methanol, acetate (ester) has been reported to have anti-inflammatory and antimicrobial activities while compound, 1-monolinoleoylglycerol trimethyl silyl ether has potential as an antiarthritic, anticancer, hepatoprotective, antimicrobial, anti-asthmatic and diuretic.

Among the identified compounds in various fractions, several secondary metabolites were common in Rp.Cme Rp.Chf, Rp.EtAc, and Rp.Bt including pentadecanoic acid, 14-methyl-, methyl ester which are previously reported for antimicrobial, antifungal and antioxidant potentials. Bioactive compound hexadecanoic acid methyl ester is reported for various pharmacological properties including inflammation, oxidative stress, depression, coagulation disorders and in the production of soaps and cosmetics [44, 45]. Some of the reported pharmacological properties of the secondary metabolites are summarized in Table S5. Another phyto-constituent, 9-Octadecenoic acid, (E)- is well known for its antioxidant, antiviral and anticancer potentials [46, 47] whereas, 9-Octadecenoic acid, 1, 2, 3-propanetriyl ester, (E, E, E) has anti-spasmodic and immuno modulating potentials [45]. Compound, 9-octadecenoic acid (Z)-, methyl ester is also reported to have anti-inflammatory, anti-androgenic, cancer preventive, dermatitigenic, hypocholesterolemic, 5-alpha reductase inhibitor, anemiagenic and insectifuge activities and anti -diarrheal activity [43, 48]. The compound, 12-Octadecenoic acid, methyl ester reported to have mosquito larval killing activity as mosquitocides [49] whereas 9,12,15-Octadecatrienoic acid, 2, 3-bis [(trimethylsilyl) oxy] propyl ester, (Z, Z, Z)- discovered in stem bark have wide range of biological activities including analgesic, anti-inflammatory, antioxidant, anti-pyretic, hypocholesterolemic, hepatoprotective and antihistaminic activities [50, 51]. Hexasiloxane, another bioactive molecule, has been reported to have antibacterial, antiseptic, skin-conditioning acidulant, acidifier, antipyretic and anti-aromatic amino acid decarboxylase properties [52, 53]. The octasiloxane reported in stem bark has antimicrobial activity [54]. Another compound, 1, 2-Benzenedicarboxylic acid, di iso octyl ester also reported to have antibacterial activity [55] whereas Bis (2-ethylhexyl) phthalate has cytotoxic and antimicrobial [56], antifungal, antioxidant, plasticizer and estrogenic activities. Phenol, 2, 4-bis(1,1-dimethylethyl) has antibacterial activity [45].

Reactive oxygen species (ROS) have been involved in the pathophysiology of a number of diseases, including carcinoma and inflammatory processes [13, 23]. Compounds from plant origin have shown antioxidant and radical scavenging potentials which neutralise free radicals that are harmful for living organisms and many other biological properties including anti-glioblastoma activity [27, 57, 58]. Free radicals have been shown to have a role in an Overloading biological systems with free radicals has a number of undesirable consequences. Proteins, lipids, and carbohydrates are among the biological components that are dominantly affected. Excessive free radicals cause oxidative damage of these vital elements, cause genetic mutations and result in cancer. Medicinal plants are rich source of antioxidant compounds thus exhibit strong free radical scavenging potentials and offer natural protection against oxidative stress induced damages. *Rhamnus pentapomica* R. Parker was investigated for its antioxidant and antitumor potentials for the first time. Our results showed that Rp.EtAc and Rp.Cme have considerable radical scavenging and anti-glioblastoma potentials (Tables 1 and 2).

The genus *Rhamnus* is widely used in folk medicine for many years owing to its varied chemical composition and active ingredients. For instance, various species of the *Rhamnus*, *Rhamnus purshiana*, *Rhamnus alaternus*, *Rhamnus kurdica, Rhamnus fallax, Rhamnus prinoides* and *Rhamnus fallax* exhibited various biological and pharmacological activities like antimicrobial, antioxidant, antiproliferative, antimutagenic, antiseptic, anti-inflammatory, laxative, anti-diabetic, skin diseases, diuretic, as a preventive for syphilis, as a depurative and antigenotoxic [59, 60]. *Rhamnus frangula* extract has tumour-inhibitory activity [7] and *Rhamnus alaternus* has anti-diabetic, anti-mutagenic and anti-genotoxic activities [61]. Because of their excellent structural chemistry, *Rhamnus pentapomica* R. Parker is a good source of minerals and phytoconstituents. It was revealed that these activities might be caused by secondary metabolites found in the many fractions [45]. For instance, 9,12,15-octadecatrienoic acid, 2, 3-bis [(trimethylsilyl) oxy]propyl ester, (Z, Z, Z)-Phenol, 2, 4-bis(1,1-dimethylethyl) [45], Bis (2-ethylhexyl) phthalate [56]. Pentadecanoic acid, 14-methyl-, methyl Ester [46, 48], 1H-Indene, 2-butyl-5-hexyloctahydro-, 4H-cyclopropa [5´,6´] benz [1´,2´,7,8] azuleno [5, 6] oxiren-4-one,8,8a- [43], 1-hexadecanol [43], 8-octadecenoic acid, methyl ester [48], (+)-3-carene, 2-à-isopropenyl- and 1, 4-methanobenzocyclodecene, 1, 2, 3, 4, 4a, 5, 8, 9, 12, 12a-decahydro found in the GC-MS analysis are reported to have antioxidant potentials [62].

Owing to the presence phytochemicals with different biological characteristics, the use of medicinal plants in cancer management and therapy has been explored. In a recent study, Rp.Cme, Rp.Chf, Rp.EtAc and Rp.Bt were tested for their anticancer efficacy against glioblastoma U87 cancer cell lines. With DMSO serving as the negative control, these experiments were carried out utilising a cell viability assay with various concentrations (25, 75, 125, 250, and 500 ug ml-1) for 24 h. The outcomes showed that Rp.Cme and Rp.EtAc have considerable cytotoxic effects against glioblastoma cells. Viability of cells treated with Rp.Cme and Rp.EtAc were reduced to 66.13% and 62.41% at concentration of 500 ug/ml respectively with IC_50_ of 147.64 μg/ml and 76.41ug/ml respectively. Compounds 17-Pentatriacontene, Phenol, 2, 4-bis (1,1-dimethylethyl)- [52], 1-Monolinoleoylglycerol trimethyl silyl ether, hexadecanoic acid, methyl ester [51] and 9-octadecenoic acid, (E)- [47] have been reported to exhibit antioxidant and anti-cancer potentials. The presence of these metabolites in the crude samples might contribute to the overall antioxidant and anti- glioblastoma effects but need further detailed studies.

## Conclusion

The current study was aimed to assess the phytochemical composition, anti-oxidant and anti- glioblastoma potentials of *Rhamnus Pentapomica* R. Parker. GC-MS analysis revealed the presence of several secondary metabolites in the crude samples. Hexadecanoic acid, methyl ester, 9-octadecenoic acid, methyl ester, 12-octadecenoic acid, methyl ester, 9, 12, and 15-octadecatrienoic acid, as well as hexasiloxane, heptasiloxane, octasiloxane, and dodecane, were abundant compounds. These samples demonstrated considerable antioxidant and anti-glioblastoma potentials. The availability of several bioactive substances in the stem bark of this plant supports its traditional use for medicinal purposes, and warrants the purification of the desired molecules and scientific exploration of their therapeutic effects.

### Supplementary Information

**Additional file 1: Table S1.** Phytochemical profile of methanol extract (Rp.Cme). **Table S2.** Phytochemical profile of chloroform fraction Rp.Chf. **Table S3.** Phytochemical profile of ethyl acetate fraction Rp.EtAc. **Table S4.** Phytochemical profile of butanol fraction Rp.Bt. **Table S5.** Bioactive compounds of stem bark of *Rhamnus pentapomica* and their biological activities.

## Data Availability

Data related to this manuscript is available to researchers from Dr. Arshad Iqbal (supervisor) upon request.
