# Supplementary material for: Phytochemical analysis, radical scavenging and glioblastoma U87 cells toxicity studies of stem bark of buckthorn (Rhamnus pentapomica R. Parker)
Source: BMC Complement Med Ther. 2024 Jan 2;24:12. doi: 10.1186/s12906-023-04309-w (PMC10759440; doi:10.1186/s12906-023-04309-w)
Supplement: Supplementary file 1 — Additional file 1: Table S1. Phytochemical profile of methanol extract (Rp.Cme). Table S2. Phytochemical profile of chloroform fraction Rp.Chf. Table S3. Phytochemical profile of ethyl acetate fraction Rp.EtAc. Table S4. Phytochemical profile of butanol fraction Rp.Bt. Table S5. Bioactive compounds of stem bark of Rhamnus pentapomica and their biological activities. [file 12906_2023_4309_MOESM1_ESM.docx]

**Supplementary files**

**Table S1**: Phytochemical profile of methanol extract (Rp.Cme)

| S. No. | Retention Time | Compound Name | Molecular Formula | Molecular Weight | | Peak area Percentage % |
| --- | --- | --- | --- | --- | --- | --- |
|  | 2.26 | 9-Octadecenoic acid, (2-phenyl-1, 3-dioxolan-4-yl) methyl ester, cis) | C_28_H_44_O_4_ | 444 | | 0.08 |
|  | 2.26 | 2, 3 bis [(trimethylsilyl) oxy] propyl ester,(Z, Z ,Z)- | C_27_H_52_O_4_Si_2_ | 496 | | 0.08 |
|  | 2.26 | 9-Octadecenoic acid, (E)- | C_18_H_34_O_2_ | 282 | | 0.08 |
|  | 3.36 | Cyclohexane, 1, 1-dimethoxy- | C_8_H_16_O_2_ | 144 |  | 4.13 |
|  | 3.36 | 3-Oxo-4-methylpentanoic acid, methyl ester, enol form | C_7_H_12_O_3_ | 144 |  | 4.13 |
|  | 3.36 | trans-3-Methyl-2-n-propyl-thiophane | C_8_H_16_S | 144 | | 4.13 |
|  | 4.97 | Oleic acid, eicosyl ester | C_38_H_74_O_2_ | 562 | | 0.03 |
|  | 4.97 | 9-Octadecenoic acid, 1, 2, 3-propanetriyl ester, (E, E, E)- | C_57_H_104_O_6_ | 884 | | 0.03 |
|  | 4.97 | 2, 3 Dihydroxypropyl elaidate (Monoelaidin) | C_21_H_40_O_4_ | 356 | | 0.03 |
|  | 7.5 | 2, 4, 6, 8, 10-Tetradecapentaenoic acid,9a-(acetyloxy) 1a, 1b, 4, 4a, 5, 7a, 7b, 8, 9, 9a-decahydro-4a, 7b-dihydroxy-3-(hydroxymethyl)-1, 1, 6, 8-tetramethyl-5-oxo-1H-cyclopropa [3,4] benz [1, 2-e] azulen-9-yl ester [1aR (1aà, 1bá, 4aá, 7aà, 7bà, 8à, 9á, 9aà)]- | C_36_H_46_O_8_ | 606 | | 0.42 |
|  | 7.5 | 1b, 4a-Epoxy-2H-cyclopenta [3, 4] cyclopr opa [8, 9] cycloundec [1, 2-b] oxiren-5 (1aH)-one, 2, 7, 9, 10-tetrakis (acetyloxy) decahydro-3, 6, 8, 8, 10a-pentamethyl- | C_28_H_38_O_11_ | 550 | | 0.42 |
|  | 7.5 | 4-Oxo-4-(1,2,2-trimethyl-5-oxocyclopen tyl)-but-2-enoic acid, methyl ester | C_13_H_18_O_4_, | 238 | | 0.42 |
|  | 9.33 | 1,4-Methanobenzocyclodecene,1,2,3,4,4a,5,8,9,12,12a-decahydro- (Tricyclopentadeca-3,7-dien[8.4.0.1(11,14)] | C_15_H_22_ | 202 | | 0.31 |
|  | 10.13 | 1H-Indene, 2-butyl-5-hexyloctahydro- | C_19_H_36_ | 264 | | 0.35 |
|  | 10.13 | 1H-Indene, 5-butyl-6-hexyloctahydro- | C_19_H_36_ | 264 | | 0.35 |
|  | 12.5 | Hexadecanoic acid, methyl ester | C_17_H_34_O_2_ | 270 | | 3.76 |
|  | 12.5 | Pentadecanoic acid, 14-methyl-, methyl Ester | C_17_H_34_O_2_ | 270 | | 3.76 |
|  | 13.38 | 4H Cyclopropa [5', 6'] benz [1', 2' : 7, 8] azuleno [5, 6-b] oxiren-4-one, 8-(acetyloxy)-1, 1a, 1b, 1c, 2a, 3, 3a, 6a, 6b, 7, 8, 8a-dodecahydro-3a, 6b, 8a-trihydroxy-2a(hydroxymethyl)-1, 1, 5, 7-tetramethyl-, [1ar (1aà, 1bá, 1cà, 2aà, 3aá, 6aà, 6bà, 7à, 8 á, 8aà)]- | C_22_H_30_O_8_ | 422 | | 0.2 |
|  | 13.31 | Dasycarpidan-1-methanol, acetate (ester) | C_20_H_26_N_2_O_2_ | 326 | | 0.2 |
|  | 13.38 | 9-Octadecenoic acid (Z)-, hexadecyl ester | C_34_H_66_O_2_ | 506 | | 0.2 |
|  | 14.16 | 9-Octadecenoic acid, methyl ester, (E)- | C_19_H_36_O_2_ | 296 |  | 7.73 |
|  | 14.16 | 9-Octadecenoic acid (Z)-, methyl ester | C_19_H_36_O_2_ | 296 |  | 7.73 |
|  | 14.16 | 12-Octadecenoic acid, methyl ester | C_19_H_36_O_2_ | 296 | | 7.73 |
|  | 15.95 | Octadecanoic acid, 9-oxo-, methyl ester | C_19_H_36_O_3_ | 312 | | 1.05 |
|  | 17.87 | 1,2-Benzenedicarboxylic acid, diisooctyl ester | C_24_H_38_O_4_ | 390 | | 0.68 |
|  | 17.87 | Bis (2-ethylhexyl) phthalate | C_24_H_38_O_4_ | 390 | | 0.68 |
|  | 20.55 | 1-Monolinoleoylglycerol trimethylsilyl Ether | C_27_H_54_O_4_Si_2_ | 498 | | 0.04 |
|  | 20.55 | 9,12,15-Octadecatrienoic acid, 2, 3-bis [(trimethylsilyl) oxy]propyl ester, (Z, Z, Z)- | C_27_H_52_O_4_Si_2_ | 496 | | 0.04 |
|  | 23.84 | Octasiloxane, 1,1,3,3,5,5,7,7,9,9,11,11,13,13,15,15-hexadecamethyl- | C_16_H_50_O_7_Si_8_ | 578 | | 5.13 |
|  | 23.84 | Hexasiloxane,1,1,3,3,5,5,7,7,9,9,11,11-dodecamethyl- | C_12_H_38_O_5_Si_6_ | 430 | | 5.13 |
|  | 23.84 | Heptasiloxane,1,1,3,3,5,5,7,7,9,9,11,11,13,13-tetradecamethyl- | C_14_H_44_O_6_Si_7_ | 504 | | 5.13 |

**Table S2:** Phytochemical profile of chloroform fraction Rp.Chf

| **S. No.** | **Retention time (min)** | **Compound’s Name** | **Molecular Formula** | **Molecular Weight** | **Peak Area %** |
| --- | --- | --- | --- | --- | --- |
|  | 2.51 | Benzene, 1,3-dimethyl- | C_8_H_10_ | 106 | 3.24 |
|  | 4.95 | Benzene, nitro- | C_6_H_5_NO_2_ | 123 | 1.26 |
|  | 8.13 | Benzenepropanoic acid, à-[(2,4-dinitrophenyl) hydrazono]-4-hydroxy- | C_15_H_12_N_4_O_7_ | 360 | 0.02 |
|  | 8.13 | Phenol, 2-cyclohexyl- | C_12_H_16_O | 176 | 0.02 |
|  | 8.13 | (+)-3-Carene, 2-à-isopropenyl- | C_13_H_20_ | 176 | 0.02 |
|  | 10.53 | Phenol, 2,4-bis(1,1-dimethylethyl)- | C_14_H_22_O | 206 | 0.05 |
|  | 12.61 | Dodecane,1-cyclopentyl-4-(3-cyclopentylpropyl)- | C_25_H_48_ | 348 | 0.02 |
|  | 12.61 | Decane,5,6-bis(2,2-dimethylpropylidene)-,(E,Z)- | C_20_H_38_ | 278 | 0.02 |
|  | 12.61 | 1H-Indene, 2-butyl-4-hexyloctahydro- | C_19_H_36_ | 264 | 0.02 |
|  | 15.58 | Eicosane | C_20_H_42_ | 282 | 0.08 |
|  | 16.54 | 8-Octadecenoic acid, methyl ester | C_19_H_36_O_2_ | 296 | 0.32 |
|  | 16.54 | 9-Octadecenoic acid, methyl ester | C19H36O2 | 296 | 0.32 |
|  | 16.54 | 9-Octadecenoic acid (Z)-, methyl ester | C19H36O2 | 296 | 0.32 |
|  | 17.5 | 1-Oxa-4,6,9,11-tetraazacyclotridecane-5,10-dithione | C_8_H_16_N_4_OS_2_ | 248 | 0.31 |
|  | 17.5 | 2H-Benz[b]oxan-3-thiocarboxamide,8-ethoxy-2-imino- | C_12_H_12_N_2_O_2_S | 248 | 0.31 |
|  | 17.5 | 1,4-Dioxa-7,9,12,14-tetraazacyclohexadecane-8,13-dithione | C_10_H_20_N_4_O_2_S_2_ | 292 | 0.31 |
|  | 20.19 | 1,2-Benzenedicarboxylic acid,diisooctyl ester | C_24_H_38_O_4_ | 390 | 0.11 |
|  | 20.19 | Bis (2-ethylhexyl) phthalate | C24H38O4 | 390 | 0.11 |
|  | 24.18 | 1-Monolinoleoylglycerol trimethylsilylether | C_27_H_54_O_4_Si_2_ | 498 | 0.01 |
|  | 26.28 | Octasiloxane,1,1,3,3,5,5,7,7,9,9,11,11,13,13,15,15-hexadecamethyl- | C_16_H_50_O_7_Si_8_ | 578 | 0.3 |
|  | 26.28 | Hexasiloxane,1,1,3,3,5,5,7,7,9,9,11,11-dodecamethyl- | C_12_H_38_O_5_Si_6_ | 430 | 0.3 |
|  | 26.28 | Heptasiloxane,1,1,3,3,5,5,7,7,9,9,11,11,13,13-tetradecamethyl- | C_14_H_44_O_6_Si_7_ | 504 | 0.3 |
|  | |  |  |  |  |

**Table S3:** Phytochemical profile of ethyl acetate fraction Rp.EtAc

| **S.No.** | **Retention time (min)** | **Compound’s Name** | **Molecular Formula** | **Molecular Weight** | **P. Area %** |
| --- | --- | --- | --- | --- | --- |
|  | 2.43 | Benzene, 1,3-dimethyl- | C_8_H_10_ | 106 | 11.29 |
|  | 6.64 | 9,12,15-Octadecatrienoic acid,2,3-bis[(trimethylsilyl)oxy]propyl ester,(Z,Z,Z)- | C_27_H_52_O_4_Si_2_ | 496 | 0.01 |
|  | 6.64 | Strychane,1-acetyl-20à-hydroxy-16-methylene- | C_21_H_26_N_2_O_2_ | 338 | 0.01 |
|  | 6.64 | 1-Monolinoleoylglycerol trimethylsilylether | C_27_H_54_O_4_Si_2_ | 498 | 0.01 |
|  | 10.1 | Butylated Hydroxytoluene | C_15_H_24_O | 220 | 0.01 |
|  | 10.1 | 4,6-di-tert-Butyl-m-cresol | C15H24O | 220 | 0.01 |
|  | 11.82 | Tetratetracontane | C_44_H_90_ | 618 | 0.03 |
|  | 11.82 | Heneicosane, 11-(1-ethylpropyl)- | C_26_H_54_ | 366 | 0.03 |
|  | 12.61 | Dodecane,1-cyclopentyl-4-(3-cyclopentylpropyl)- | C_25_H_48_ | 348 | 0.02 |
|  | 12.61 | 22-Tricosenoic acid | C_23_H_44_O_2_ | 352 | 0.02 |
|  | 12.61 | á-d-Mannofuranose,2,3:5,6-di-O-ethylboranediyl-1-O-(10-undecen-1-yl)- | C_21_H_38_B_2_O_6_ | 408 | 0.02 |
|  | 15.3 | Dibutyl phthalate | C_16_H_22_O_4_ | 278 | 0.12 |
|  | 15.3 | 1,2-Benzenedicarboxylic acid, butyl2-methylpropyl ester | C16H22O4 | 278 | 0.12 |
|  | 16.54 | 9-Octadecenoic acid (Z)-, methyl ester | C_19_H_36_O_2_ | 296 | 0.34 |
|  | 16.54 | 9-Octadecenoic acid, methyl ester | C19H36O2 | 296 | 0.34 |
|  | 16.54 | 8-Octadecenoic acid, methyl ester | C19H36O2 | 296 | 0.34 |
|  | 16.77 | 1,2-Benzenedicarboxylic acid,diisooctyl ester | C_26_H_54_ | 366 | 0.04 |
|  | 16.77 | 7-Hexadecenal, (Z)- | C_16_H_30_O | 238 | 0.04 |
|  | 16.77 | Cholest-22-ene-21-ol,3,5-dehydro-6-methoxy-, pivalate | C_33_H_54_O_3_ | 498 | 0.04 |
|  | 19.01 | 17-Pentatriacontene | C_35_H_70_ | 490 | 0.01 |
|  | 19.01 | 9,12,15-Octadecatrienoic acid, 2,3-bis[(trimethylsilyl)oxy]propyl ester,(Z,Z,Z)- | C_27_H_52_O_4_Si_2_ | 496 | 0.01 |
|  | 19.01 | Octadecane, 3-ethyl-5-(2-ethylbutyl)- | C_26_H_54_ | 366 | 0.01 |
|  | 25.59 | Octasiloxane,1,1,3,3,5,5,7,7,9,9,11,11,13,13,15,15-hexadecamethyl- | C_16_H_50_O_7_Si_8_ | 578 | 0.33 |
|  | 25.59 | Hexasiloxane,1,1,3,3,5,5,7,7,9,9,11,11-dodecamethyl- | C_12_H_38_O_5_Si_6_ | 430 | 0.33 |

**Table S4:** Phytochemical profile of butanol fraction Rp.Bt

| **S. No.** | **Retention time (min)** | **Compound’s Name** | **Molecular Formula** | **Molecular Weight** | **Peak Area %** |
| --- | --- | --- | --- | --- | --- |
|  | 2.36 | 3-Hexanone, 2,5-dimethyl-4-nitro- | C_8_H_15_NO_3_ | 173 | 0.01 |
|  | 2.36 | Pentanoic acid, 2,2,4-trimethyl-3-hydroxy-,isobutyl Ester | C_12_H_24_O_3_ | 216 | 0.01 |
|  | 2.36 | Pentane, 1-bromo-3,4-dimethyl- | C_7_H_15_Br | 178 | 0.01 |
|  | 11.84 | 1,4-Methanobenzocyclodecene,1,2,3,4,4a,5,8,9,12,12a-decahydro | C_15_H_22_ | 202 | 0.01 |
|  | 12.61 | 1H-Indene, 2-butyl-5-hexyloctahydro- | C_19_H_36_ | 264 | 0.01 |
|  | 12.61 | Decane, 5,6-bis(2,2 dimethylpropylidene)-,(E,Z)- | C_20_H_38_ | 278 | 0.01 |
|  | 12.61 | Dodecane, 1-cyclopentyl-4-(3-cyclopentylpropyl)- | C_25_H_48_ | 343 | 0.01 |
|  | 14.94 | Pentadecanoic acid, 14-methyl-, methyl Ester | C_17_H_34_O_2_ | 270 | 0.02 |
|  | 14.94 | Hexadecanoic acid, methyl ester | C_17_H_34_O_2_ | 270 | 0.02 |
|  | 14.94 | Hexadecanoic acid, methyl ester | C_17_H_34_O_2_ | 270 | 0.02 |
|  | 16.56 | 8-Octadecenoic acid, methyl ester | C_19_H_36_O_2_ | 296 | 0.06 |
|  | 16.56 | 9-Octadecenoic acid, methyl ester | C_19_H_36_O_2_ | 296 | 0.06 |
|  | 16.56 | 11-Octadecenoic acid, methyl ester, (Z)- | C_19_H_36_O_2_ | 296 | 0.06 |
|  | 20.21 | 1,2-Benzenedicarboxylic acid,diisooctyl ester | C_24_H_38_O_4_ | 390 | 0.06 |
|  | 20.21 | Bis (2-ethylhexyl) phthalate | C_24_H_38_O_4_ | 390 | 0.06 |
|  | 26.13 | Hexasiloxane,1,1,3,3,5,5,7,7,9,9,11,11-dodecamethyl- | C_12_H_38_OSi_6_ | 430 | 0.09 |
|  | 26.13 | Octasiloxane,1,1,3,3,5,5,7,7,9,9,11,11,13,13,15,15-hexadecamethyl- | C_16_H_50_O_7_Si_8_ | 578 | 0.09 |
|  | 26.13 | Heptasiloxane,1,1,3,3,5,5,7,7,9,9,11,11,13,13-tetradeca methyl- | C_14_H_44_O_6_Si_7_ | 504 | 0.09 |

**Table S5** Bioactive compounds of stem bark of *Rhamnus pentapomica* and their biological activities

| **S. No.** | **Name of the Compound** | **Compound Nature** | **Pharmacological Activities** |
| --- | --- | --- | --- |
|  | Pentadecanoic acid, 14-methyl-, methyl Ester | Palmitic acid methyl ester | Antimicrobial, antifungal, Antioxidant (Beschi, *et al.,*2021; Elaiyaraja, and Chandramohan, 2018). |
|  | Pentanoic acid, 2, 2, 4-tri methyl-3-hydroxy-, iso butyl Ester | Fatty acid ester | No activity found |
|  | Hexadecanoic acid, methyl ester | Palmitic acid ester | Anti-oxidant, Anti-inflammatory, anti-uric acid, hemolytic, lubricant, nematicide, anti-alopecic, antidepressant and also used in cosmetic industry (Maje. 2021). |
|  | Octadecanoic acid, 9-oxo-, methyl ester | Stearic acid | Lower LDL cholesterol Beschi *et al.,* 2021). |
|  | 9-Octadecenoic acid, (E)- | Fatty acid | Antioxidant, antiviral and anticancer (Reza *et al.,* 2021). |
|  | 9-Octadecenoic acid, (2-phenyl-1, 3-dioxolan-4-yl) methyl ester, cis) | Fatty acid ester | No activity reported |
|  | 8-Octadecenoic acid, methyl ester | Fatty acid ester | Antioxidant and antimicrobial activities (Elaiyaraja, and Chandramohan, 2018). |
|  | 9-Octadecenoic acid, methyl ester | Fatty acid ester | Anti-oxidant, anticancer (Beschi *et al.,* 2021). |
|  | 9-Octadecenoic acid (Z)-, hexa decyl ester (Oleic acid, hexadecyl ester/ oleate) | Oleic acid | Anti-inflammatory Anemiagenic, Dermatitigenic Choleretic (Varadharajan *et a.,l* 2016). |
|  | 9-Octadecenoic acid, methyl ester, (E)- | Fatty acid Ester | No activity reported |
|  | 9-Octadecenoic acid (Z)-, methyl ester | Fatty acid Ester | Anti-inflammatory, antiandrogenic , cancer preventive , dermatitigenic, hypocholesterolemic, 5-alpha reductase inhibitor, anemiagenic and insectifuge activities (Elaiyaraja, and Chandramohan, 2018). |
|  | 9,12,15-Octadecatrienoic acid, 2, 3-bis [(trimethylsilyl) oxy]propyl ester, (Z, Z, Z)- | Fatty acid ester | Anti-nociceptive, aesthetics, anticonvulsant, against inflammation, anti-oxidant, against pyrexia, bacteriostatic, antiproleferataive, hypocholesterolemic, hepato-protective, nematicide, antihistaminic and in Covid-19 complications (Chirumamilla *et al*., 2022). |
|  | 11-Octadecenoic acid, methyl ester, (Z)- | Fatty acid ester | Antioxidant, antiviral and anticancer (Reza *et al.,* 2021). |
|  | 12-Octadecenoic acid, methyl ester | Fatty acid ester | Mosquito larval killing activity, mosquitocides (Baranitharan *et al.,* 2019). |
|  | Oleic acid, eicosyl ester | Oleic acid | Anti-inflammatory Anemiagenic, Dermatitigenic Choleretic (Varadharajan, *et al* 2016). |
|  | 9-Octadecenoic acid, 1, 2, 3-propanetriyl ester, (E, E, E)- | Fatty acid ester | Antipyretic, acidifier, increase aromatic amino acid decarboxylase activity, catechol-o-methyl-transferase inhibitor, decrease glutamate oxaloacetate transaminase (Owolabi *et al.,* 2018). |
|  | 2, 4, 6, 8, 10-Tetradecapentaenoic acid,9a-(acetyloxy) 1a, 1b, 4, 4a, 5, 7a, 7b, 8, 9, 9a-decahydro-4a, 7b-dihydroxy-3-(hydroxymethyl)-1, 1, 6, 8-tetramethyl-5-oxo-1H-cyclopropa [3, 4] benz [1, 2-e] azulen-9-yl ester [1aR (1aà, 1bá, 4aá, 7aà, 7bà, 8à, 9á, 9aà)]- | - | No activity found |
|  | 1b, 4a-Epoxy-2H-cyclopenta [3, 4] cyclopr opa [8, 9] cycloundec [1, 2-b] oxiren-5 (1aH)-one, 2, 7, 9, 10-tetrakis (acetyloxy) decahydro-3, 6, 8, 8, 10a-pentamethyl- |  |  |
|  | 1H-Indene, 2-butyl-5-hexyloctahydro- | _ | antioxidant, antibacterial, and anti-inflammatory properties which promote wound healing (Fridayanti *et al.,* 2022) |
|  |  |  |  |
|  | 1H-Indene, 5-butyl-6-hexyloctahydro- |  | Not found |
|  | 4H Cyclopropa [5', 6'] benz [1', 2' : 7, 8] azuleno [5, 6-b] oxiren-4-one, 8-(acetyloxy)-1, 1a, 1b, 1c, 2a, 3, 3a, 6a, 6b, 7, 8, 8a-dodecahydro-3a, 6b, 8a-trihydroxy-2a(hydroxymethyl)-1, 1, 5, 7-tetramethyl-, [1ar (1aà, 1bá, 1cà, 2aà, 3aá, 6aà, 6bà, 7à, 8 á, 8aà)]- | - | Antibacterial and antioxidant activity (Madhavan, 2021). |
|  | Dasycarpidan-1-methanol, acetate (ester) | An alkaloid | Anti-inflammatory, antimicrobial (Alqahtani *et al.,* 2022; Al and Mohammed. 2016) |
|  |  |  |  |
|  | 1,2-Benzenedicarboxylic acid, diisooctyl ester | Phthalic ester | Antibacterial (Análise. 2022) |
|  |  |  |  |
|  | 1,2-Benzenedicarboxylic acid, butyl2-methylpropyl ester | Phthalic ester | Antimicrobial activity (Beschi, *et al.,* 2021), used in preparation of perfumes and cosmetics (Krishnamoorthy and Subramaniam, 2014). |
|  | Bis (2-ethylhexyl) phthalate | - | Cytotoxic and antimicrobial activity (Habib Karim, 2009). |
|  | 1-Monolinoleoylglycerol trimethylsilyl Ether |  |  |
|  | Octasiloxane, 1, 1, 3, 3, 5, 5, 7, 7, 9, 9, 11, 11, 13, 13, 15, 15-hexadecamethyl- | Lactone | Antimicrobial (Falowo *et al.,* 2017). |
|  | Hexasiloxane, 1, 1, 3, 3, 5, 5, 7, 7, 9, 9, 11, 11-dodecamethyl- | Siloxane derivative | Antimicrobial, Antiseptic, Hair Conditioning Agent, Skin- Conditioning Agent-Emollient; Solvent (Mary and Giri 2016; Diab *et al.,* 2021). |
|  | Heptasiloxane, 1, 1, 3, 3, 5, 5, 7, 7, 9, 9, 11, 11, 13, 13-tetradecamethyl- | Siloxane derivative | Acidulant, Acidifier, control of fever, increase aromatic amino acid decarboxylase activity, anti- uric acid, urinary-acidulant (Owolabi *et al.,* 2018) |
|  | Benzene, 1, 3-dimethyl- | Benzene | Benzene, 1, 3-dimethyl has Antimicrobial, anti-inflammatory (Madhavan, 2021). |
|  | Benzene, nitro- | Benzene | No activity found |
|  | Benzenepropanoic acid, à-[(2, 4-dinitrophenyl)hydrazono]-4-hydroxy- | Benzene | No activity found |
|  | Phenol, 2-cyclohexyl- | Phenol | Antiviral (Li *et al.,* 2021). |
|  |  |  |  |
|  | (+)-3-Carene, 2-à-isopropenyl- | Terpenoids | Antifungal activity (Kamg *et al.,*2019). Anti-feedant, antioxidant (Krishnamoorthy and Subramaniam 2014). |
|  | Phenol, 2, 4-bis(1, 1-dimethylethyl)- | Phenolic compound | Antimicrobial, Anesthetic, Anti-oxidant, Antiseptic, Cancer preventive, Pesticide, Fungicide (Mary and Giri 2016). |
|  | Dodecane,1-cyclopentyl-4-(3-cyclopentylpropyl)- | Hydrocarbon | No activity reported |
|  | Decane,5, 6-bis(2, 2-dimethylpropylidene)-,(E, Z)- | Hydrocarbon | Antibacterial (Nahar, *et al.,* 2016). |
|  | Octadecane, 3-ethyl-5-(2-ethylbutyl)- | an alkane derivative | Antimicrobial activity (Alqahtani *et al.,* 2022). |
|  | 7-Hexadecenal, (Z)- | Aldehyde | Anti-viral (Shehata *et al.,* 2019) against cancer (Raval *et al.* 2016); inhibit free radical production, anti-fungal and insecticidal activities (Sathya *et al.* 2016). |
|  | Eicosane | Aliphatic Hydrocarbon | Anti-inflammatory, analgesic and antipyretic effects (Okechukwu. 2020) |
|  | 1-Oxa-4, 6, 9, 11-tetraazacyclotridecane-5, 10-dithione | - | No activity found |
|  | 1, 4-Dioxa-7, 9, 12, 14-tetraazacyclohexadecane-8, 13-dithione | - | No activity found |
|  | 2H-Benz[b]oxan-3-thiocarboxamide,8-ethoxy-2-imino- | - | No activity found |
|  | 1-Monolinoleoylglycerol trimethyl silyl ether | Steroid | Antiarthritic, Anticancer, Hepatoprotective, |
|  |  |  | Antimicrobial, Antiasthma, Diuretic (Tyagi and Agarwal. 2017). |
|  | Strychane,1-acetyl-20à-hydroxy-16-methylene- | Strychnos alkaloids | No activity found |
|  | Butylated Hydroxytoluene | Derivatives of phenol | Anti-oxidant and Anticancer activity (Guarisco *et al.,* 2008). |
|  | 4, 6-di-tert-Butyl-m-cresol | Phenole | Antimicrobial, Anesthetic, Anti-oxidant, Antiseptic, Cancer preventive, Pesticide, Fungicide (Mary and Giri 2016). |
|  | Tetratetracontane | Aliphatic hydrocarbon | Antioxidant and cytoprotective activities (Jayalakshmi *et al.,* 2018). |
|  | Heneicosane, 11-(1-ethylpropyl)- | Branched alkanes | Antimicrobial (Vanitha *et al.,* 2020). |
|  | 22-Tricosenoic acid | Saturated Fattyacid | Antiviral and cytotoxic (Rashid *et al.,* 2018). Gollo *et al.,* 2020). Act as lipid anchor in bio membranes |
|  | á-d-Mannofuranose, 2,3 : 5, 6-di-O-ethylboranediyl-1-O-(10-undecen-1-yl)- | Carbohydrates | No activity reported |
|  | Dibutyl phthalate | Fatty acid | Inhibit microbial growth, antifouling, used in perfumes nail polish, and ectoparasitic agent (Maje. 2021; Chirumamilla *et al*., 2022). |
|  | Cholest-22-ene-21-ol, 3, 5-dehydro-6-methoxy-, pivalate | Steroid | Antimicrobial (Waheed *et al.,* 2019) |
|  |  |  | antimicrobial, anti-inflammatory, antiarthritic and anti-asthmatic activities (TK and Khaleel. 2021). |
|  | 17-Pentatriacontene | Unsaturated aliphatic hydrocarbons | Anti-inflammatory Anticancer Antibacterial Antiarthritic (Kumar *et al.,* 2018) |
|  | 3-Hexanone, 2,5-dimethyl-4-nitro- | Ketone | No activity found |
|  | Pentane, 1-bromo-3,4-dimethyl- | Organobromides | No activity found |
|  | 4-Oxo-4-(1, 2, 2-trimethyl-5-oxocyclopen tyl)-but-2-enoic acid, methyl ester | Fumaranilic acid | No activity found |
|  | 3-Oxo-4-methylpentanoic acid, methyl ester, enol form | - | No activity found |
|  | 1, 4-Methanobenzocyclodecene, 1, 2, 3, 4, 4a, 5, 8, 9, 12, 12a-decahydro- | - | Antioxidant, analgesic and antidiarrheal activity (Islam *et al .,* 2022). |
|  | Cyclohexane, 1, 1-dimethoxy- | Cyclohexane | Antibacterial (Subramaniam *et al*., 2012). |
|  | trans-3-Methyl-2-n-propyl-thiophane | Hydrocarbon | No activity found |
|  | 2, 3 bis [(trimethylsilyl) oxy] propyl ester,(Z, Z ,Z)- | Fatty acid ester | Antimicrobial and antioxidant activity (Imad *et al .,* 2015). |
|  | 2, 3 Dihydroxypropyl elaidate (Monoelaidin) | - | Antimicrobial, antihypertensive, antioxidant and anticancer (Adeyemo *et al.,* 2021; antitumor activity (บุษราคัม สิงห์ ชัย and อัจฉรา พรรณ บุญ สิทธิ์, 2019). |
